# Targeting Chemokine—Glycosaminoglycan Interactions to Inhibit Inflammation

**DOI:** 10.3389/fimmu.2020.00483

**Published:** 2020-03-31

**Authors:** Helena Crijns, Vincent Vanheule, Paul Proost

**Affiliations:** Laboratory of Molecular Immunology, Department of Microbiology, Immunology and Transplantation, Rega Institute for Medical Research, KU Leuven, Leuven, Belgium

**Keywords:** chemokine, chemotaxis, heparin, heparan sulfate, leukocyte migration

## Abstract

Leukocyte migration into tissues depends on the activity of chemokines that form concentration gradients to guide leukocytes to a specific site. Interaction of chemokines with their specific G protein-coupled receptors (GPCRs) on leukocytes induces leukocyte adhesion to the endothelial cells, followed by extravasation of the leukocytes and subsequent directed migration along the chemotactic gradient. Interaction of chemokines with glycosaminoglycans (GAGs) is crucial for extravasation *in vivo*. Chemokines need to interact with GAGs on endothelial cells and in the extracellular matrix in tissues in order to be presented on the endothelium of blood vessels and to create a concentration gradient. Local chemokine retention establishes a chemokine gradient and prevents diffusion and degradation. During the last two decades, research aiming at reducing chemokine activity mainly focused on the identification of inhibitors of the interaction between chemokines and their cognate GPCRs. This approach only resulted in limited success. However, an alternative strategy, targeting chemokine-GAG interactions, may be a promising approach to inhibit chemokine activity and inflammation. On this line, proteins derived from viruses and parasites that bind chemokines or GAGs may have the potential to interfere with chemokine-GAG interactions. Alternatively, chemokine mimetics, including truncated chemokines and mutant chemokines, can compete with chemokines for binding to GAGs. Such truncated or mutated chemokines are characterized by a strong binding affinity for GAGs and abrogated binding to their chemokine receptors. Finally, Spiegelmers that mask the GAG-binding site on chemokines, thereby preventing chemokine-GAG interactions, were developed. In this review, the importance of GAGs for chemokine activity *in vivo* and strategies that could be employed to target chemokine-GAG interactions will be discussed in the context of inflammation.

## Introduction

Chemotactic cytokines or chemokines, complement fragments C3a and C5a, bioactive lipids such as leukotrienes, and formylated peptides interact with specific G protein-coupled receptors (GPCRs) on leukocytes and are predominant mediators of leukocyte migration to an inflammatory site (1). Chemokines constitute a family of about 50 small, mostly secreted proteins comprising between 60 and 90 amino acids (2, 3). Chemokines are the only group of cytokines that interact with GPCRs (4, 5). In contrast to other chemoattractants, chemokines are characterized by their specificity for leukocyte subsets (5). Accordingly, chemokine receptors are expressed on different groups of leukocytes in a cell-specific manner (3, 6). Pathogen-associated molecular patterns (PAMPs), derived from an infectious microorganism, can directly induce the production of chemokines through pattern recognition receptors (PRRs) by tissue-resident immune cells, including macrophages, and numerous parenchymal and stromal cells. In addition, chemokine production can be caused by endogenous molecules associated with injury or infection, including defensins and elastase, and by signaling of danger molecules through PRRs (7). Binding of locally produced chemokines to their chemokine receptors induces leukocyte adhesion to the endothelial cells, followed by extravasation of the leukocytes and subsequent directed migration to the site of inflammation (2, 3). To be exposed on the endothelial layer of blood vessels and to create a concentration gradient, chemokines need to bind to glycosaminoglycans (GAGs) such as heparan sulfate (HS) on endothelial cells and in tissues (8–10). In addition to regulating leukocyte trafficking, chemokines play a role in cell survival, effector responses such as degranulation and the coordination of recirculation and homing of lymphocytes. However, the function of chemokines is not restricted to leukocyte physiology alone, since they contribute to several processes such as tumor growth and metastasis, haematopoiesis, angiogenesis, and organogenesis (3, 5, 11).

Chemokines can be classified into functional groups. Inflammatory chemokines are involved in the recruitment of effector leukocytes to the site of inflammation. They are induced upon infection, inflammation, tissue injury, tumors or other stress factors. Examples of inflammatory chemokines include CXCL1-3, CXCL5-6, and CXCL8, which regulate neutrophil recruitment. Homeostatic chemokines, by contrast are constitutively expressed and regulate basal leukocyte migration. An example of a homeostatic chemokine is CCL27, which plays a role in skin homing of T cells. Some chemokines demonstrate both inflammatory and homeostatic activities, hence they are referred to as dual-function chemokines (3, 11–13). These include CXCL12, which is important for the retention of neutrophils in the bone marrow (BM), and also synergizes with other chemoattractants to attract inflammatory cells (14).

Alternatively, chemokines can be classified based on their structure according to a conserved tetra-cysteine motif that forms two disulphide bridges and that determines the specific tertiary chemokine structure. Four subfamilies can be defined based on the position of the two NH_2_-terminal cysteine residues. In the CC chemokine subgroup, the two first cysteine residues are adjacent, whereas these residues are separated by one or three amino acids in the CXC and CX3C chemokine subfamilies, respectively. C chemokines are an exception, since they lack two conserved cysteine residues (3, 7, 12). The CXC chemokines can be subdivided in either ELR^+^ or ELR^−^ CXC chemokines. ELR^+^ CXC chemokines include a Glu-Leu-Arg amino acid sequence preceding the CXC sequence and are neutrophil attractants with angiogenic activity (3, 15). CXC chemokines lacking the ELR motif that bind to CXC chemokine receptor 3 (CXCR3) act on natural killer (NK) cells and activated T lymphocytes and display angiostatic activity. Members of this group include CXCL4, CXCL4L1, CXCL9, CXCL10, and CXCL11 (16). Although chemokines demonstrate low amino acid sequence homology, their tertiary structure is characterized by remarkable similarities (3).

## Two Main Interaction Partners of Chemokines: Chemokine Receptors and Glycosaminoglycans

### Chemokines Receptors

GPCRs with seven transmembrane domains mediate the recognition of chemokine-encoded messages (7). These GPCRs comprise a polypeptide chain with three intracellular and three extracellular loops, a serine/threonine-rich intracellular COOH-terminal and an acidic NH_2_-terminal extracellular domain. Receptor signaling and internalization is mediated by the transmembrane domains, cytoplasmic loops and COOH-terminal domain. The NH_2_-terminal domain and a pocket created by the transmembrane domains and extracellular loops are involved in ligand recognition (1). A unique structural feature of the chemokine receptors is the DRYLAIV amino acid sequence present in the second intracellular loop domain, which is required for efficient coupling with G proteins of the G_α*i*_ class (5, 12). The chemokine receptors are classified into four subfamilies in accordance with the cysteine motifs of their main ligands: CXCR, CCR, CX3CR, and XCR (1, 12). Upon binding of chemokines, chemokine receptors undergo conformational changes giving rise to the activation of intracellular effectors via G proteins and/or β-arrestins, initiating signal transduction pathways and cellular responses (17–23).

In addition, several atypical chemokine receptors (ACKRs) have been identified (6, 24). These atypical receptors are characterized by a modified or lacking DRYLAIV motif, resulting in the inability of eliciting conventional G protein-coupled signaling processes. The ACKRs influence the internalization and function of chemokines through interaction with β-arrestin signaling pathways. They regulate inflammatory and immune responses by functioning as scavenger or decoy receptors or chemokine transporters (1, 3, 12).

Most inflammatory chemokines bind to several receptors and most chemokine receptors recognize multiple ligands. This binding promiscuity is characteristic for the chemokine network (3, 5). Thus, the chemokine/chemokine receptor network seems highly redundant (25). However, this functional redundancy is not absolute (26, 27). It has been suggested that chemokines are under temporal and spatial control *in vivo*, and that the localization and timing determine a different biological outcome in different tissues. To ensure appropriate inflammatory responses and to avoid undesirable inflammation, this complex system must be tightly controlled, thereby enabling fine-tuning of leukocyte responses to different inflammatory stimuli. The mechanisms which regulate the interactions between chemokines and chemokine receptors, including down-regulation of chemokine activity by atypical receptors, alternative signaling responses and posttranslational modifications (PTMs), have recently been reviewed (28–30). In contrast to the originally expected redundancy of the chemokine network, recent work demonstrates extreme specificity of the chemokine/chemokine receptor system. Girbl et al., identified distinct and non-redundant roles for two murine CXCR2 ligands CXCL1 and CXCL2 in neutrophil transendothelial migration (31). In addition, Coombs et al. revealed that differential trafficking of the chemokine receptors CXCR1 and CXCR2 regulates neutrophil clustering and dispersal at sites of tissue damage in zebrafish (32). Furthermore, Dyer et al. provide evidence for both redundancy and specificity of the chemokine receptors CCR1, CCR2, CCR3 and CCR5 dependent on the context (33).

The interactions of chemokines and chemokine receptors were traditionally described by a two-step/two-site mechanism (34–36). In the spatial formulation (i.e., two-site), the NH_2_-terminus of the receptor recognizes the chemokine globular core (site 1 interaction), followed by the insertion of the unstructured chemokine NH_2_-terminus into the receptor transmembrane bundle (site 2 interaction). In the functional formulation (i.e., two-step), site 1 provides affinity and specificity, whereas site 2 elicits receptor activation. With this knowledge, it is not surprising that minor modifications at the NH_2_-terminus of chemokines may have profound effects on their activity. However, more and more evidence supports a more complex model (multiple steps/multiple binding sites in the interaction of chemokines and their receptors) to mediate increasingly diverse outcomes (37). The new paradigms in chemokine receptor signal transduction have recently been reviewed by Kleist et al. (38). These authors indicate that we should move beyond the two-site model, since chemokine receptor signaling is influenced by PTMs of chemokine receptors, chemokine, and chemokine receptor dimerization and endogenous non-chemokine ligands.

### Glycosaminoglycans (GAGs)

**Figure 1:**
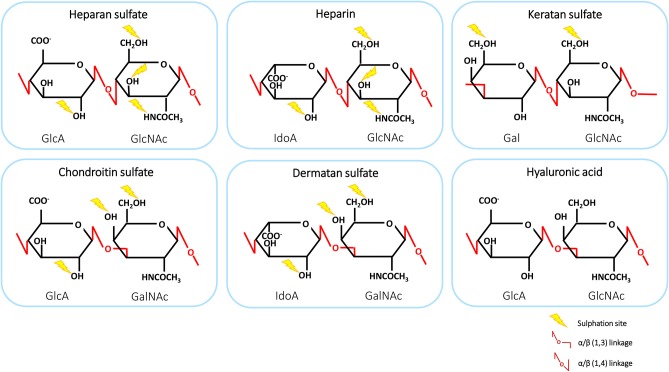
The structure and disaccharide composition of glycosaminoglycans (GAGs). The backbone of GAGs consists of repeating disaccharide subunits, composed of uronic acid or galactose and an amino sugar. Linkages are shown in red and sites of sulphation are indicated by yellow lightning bolts. GlcA, D-glucuronic acid; GlcNAc, N-acetyl-D-glucosamine; GalNAc, N-acetyl-D-galactosamine; Gal, D-galactose; IdoA, L-iduronic acid.

GAGs are negatively charged, linear polysaccharides comprising repeated disaccharide units, varying in basic composition of the saccharide, linkage, and patterns of acetylation and N- and O-sulphation. The structures of GAGs are highly variable in composition and length, ranging from 1 to 25,000 disaccharide units. Therefore, these polysaccharides exhibit the largest diversity among biological macromolecules (8, 39). GAGs interact with a wide variety of proteins, including proteases, growth factors, cytokines, chemokines and adhesion molecules, enabling them to participate in physiological processes, such as protein function, cellular adhesion and signaling (9, 40). GAGs can be classified into six groups: heparan sulfate (HS), heparin, chondroitin sulfate (CS), dermatan sulfate (DS), keratan sulfate (KS), and hyaluronic acid (HA) (39). The structures and disaccharide composition of GAGs are shown in Figure 1. The disaccharide subunits are composed of an amino sugar residue [N-acetyl-D-galactosamine (GalNAc) or N-acetyl-D-glucosamine (GlcNAc)] and an uronic acid residue [D-glucuronic acid (GlcA) or L-iduronic acid (IdoA)] or D-galactose (Gal) (41, 42). Interestingly, HS has a multidomain structure with sulphated IdoA-containing domains or NS-domains (usually 5-10 disaccharides) separated by flexible spacers of low sulphation that have an acetylated GlcA-GlcNAc sequence (43). HS proteoglycans (HSPGs) account for 50 to 90% of total endothelial proteoglycans (PGs) (44). GAGs, including heparin and HA, can be present in plasma as soluble molecules. Alternatively, GAGs are encountered in surface-bound forms as PGs (8, 39). GAGs, other than heparin and HA, are frequently found covalently attached to protein cores, thereby forming PGs (9). These structures are ubiquitously present on cell surfaces as well as in the extracellular matrix (ECM). There, the PGs serve as a macromolecular coating, also known as glycocalyx, which can interact with proteins such as chemokines (8).

Chemokine binding to GAGs is required for chemokine-induced leukocyte migration *in vivo*. Mutants demonstrating impaired GAG-binding capacity retained the ability to induce chemotaxis *in vitro*, but failed to elicit cell migration *in vivo* (45–49). Chemokines interact with GAGs of the ECM and endothelial cell surfaces (39, 45, 50, 51). Immobilization of chemokines enables the formation of a chemokine gradient, which is indispensable for leukocyte recruitment. This tethering mechanism prevents the diffusion of the chemokines in the blood stream and facilitates localized high concentrations of chemokines that are produced (39, 45). Furthermore, GAGs may play a role in the abluminal-to-luminal transcytosis of chemokines (52, 53). In addition, GAGs may protect chemokines against proteolysis and may influence chemokine-GPCR signaling, thereby regulating chemokine function (9, 54–56).

## The Importance of Chemokine-Glycosaminoglycan Interactions

### Leukocyte Extravasation, Gradient Formation, and Transcytosis of Chemokines

A hallmark of immune cell trafficking at sites of inflammation and in normal immune surveillance is the migration of leukocytes from the circulation across the endothelium. Therefore, leukocytes need to adhere to the luminal surface of the endothelium. As an inflammatory response develops, cytokines and other inflammatory mediators stimulate the local expression of cell adhesion molecules. First, leukocytes attach to the endothelium by a low-affinity interaction between selectins on the endothelium and their carbohydrate counter-ligands mediating leukocyte tethering and rolling (52, 57–60). In this way, chemokines are able to bind to their leukocyte-specific chemokine receptor(s) resulting in the activation of integrins on the leukocyte. The interaction between the leukocyte integrins and their ligands, such as immunoglobulin-like intercellular adhesion molecules, mediates firm adhesion to the endothelium, enabling the leukocyte to force its way between endothelial cells (52). During this transendothelial migration, the leukocyte squeezes in between two neighboring endothelial cells without disrupting the integrity of the endothelial barrier (61). For neutrophils, this is accomplished by homotypic binding of platelet endothelial cell adhesion molecule-1 (PECAM-1) on the neutrophil with PECAM-1 within the endothelial junction (62). Moreover, it has been shown that PECAM-1 is able to bind heparin and HS by a site that is distinct from that required for haemophilic binding (63). In addition, leukocytes were shown to migrate through endothelial cells (64–66). This process of transcellular migration involves many of the same molecules and mechanisms that regulate paracellular migration.

To ensure the directional guidance of leukocytes across the endothelium and through the ECM into the tissue, a chemoattractant gradient is necessary. However, soluble chemokine gradients cannot persist on the luminal endothelial surface, since they are disturbed by the blood flow (67–69). In addition, soluble chemoattractant gradients would activate leukocytes in the circulation prior to their selectin-mediated adhesive interaction with the endothelium, resulting in the loss of leukocytes' ability to initiate adhesion and emigration (70). Therefore, it has been proposed that chemokines that are bound or immobilized on the luminal endothelial surface more effectively promote leukocyte adhesion to the endothelium and subsequent migration. A first proof of this haptotaxis was the *in situ* binding of CXCL8 to endothelial cells of venules and veins in human skin and the ability of immobilized CXCL8 to induce *in vitro* neutrophil migration (71, 72). In addition, chemokines undergo transcytosis through the endothelium and are presented at the luminal surface to adherent leukocytes. Both CXCL8 and CCL5 were bound at the abluminal surface of the endothelium, internalized into caveolae and transported transcellularly to the luminal surface (57). It has even been shown that a COOH-terminally truncated CXCL8 analog with impaired heparin binding and impaired immobilization on endothelial HS was unable to be transcytosed and lost its capacity to induce neutrophil migration *in vitro* and *in vivo* (57). Because many of the cell types that produce chemokines are extravascular, chemokine transcytosis and presentation by the endothelium provides a mechanism where through chemokines can stimulate leukocyte emigration (73). A similar mechanism has been shown for CCL19 in the high endothelial venules of lymphoid tissues where it mediates T cell recruitment and suggests a role for this mechanism in normal immune surveillance (74). Noteworthy, endothelial cells also produce chemokines themselves, which are stored in Weibel-Palade bodies and do not need to be transcytosed (73, 75).

In addition, ACKR1 or the duffy antigen receptor for chemokines (DARC), expressed on red blood cells and endothelial cells of postcapillary venules, was shown to bind chemokines, such as CXCL1, CXCL8, CCL2, and CCL5, in inflamed and normal human tissues (76, 77). Mice with targeted disruption of the ACKR1 gene show no developmental abnormalities, but show increased inflammatory infiltrates in lung, liver and/or peritoneum when challenged with lipopolysaccharide (LPS) and/or thioglycolate (78, 79). These data suggest that the intensity of inflammatory reactions is modulated by ACKR1 and that ACKR1 acts as a sink for chemokines. Endothelial ACKR1 may also play a role in chemokine transcytosis in endothelial cells, since it is localized to endothelial caveolae and binds and internalizes chemokines (80, 81). Moreover, it has been suggested that ACKR1 acts as a chemokine-presenting molecule on the endothelium (82). Girbl et al. revealed a self-guided migration response of transmigrating neutrophils. More specifically, neutrophil-derived CXCL2 was presented on ACKR1 at endothelial junctions, thereby enabling unidirectional, paracellular transendothelial migration of neutrophils *in vivo* (31). However, chemokines bound to ACKR1 on red blood cells do not activate neutrophils anymore (82). Therefore, GAGs may be more important in chemokine presentation.

Although very difficult to prove *in vivo*, chemoattractant gradient formation has been reported in tissues and venules. Recently, chemokines were shown to localize within postcapillary venules in a GAG-dependent way. For example, localized extravascular release of CXCL2 induced directed migration of neutrophils along a haptotactic gradient on the endothelium toward the tissue as visualized by intravital microscopy (50). This sequestration of chemokines occurred only in venules and was HS-dependent. Transgenic mice overexpressing heparanase showed altered and random crawling of neutrophils in response to CXCL2, which was translated into a decreased number of emigrated neutrophils. In addition, fluorescently labeled CXCL8 formed an extracellular gradient in zebrafish tissue that decays within a distance of 50–100 μm from the producing cells and that was immobilized on HSPGs on the local venous vasculature (51). Inhibition of this interaction compromised both directional guidance and restriction of neutrophil motility. This suggests that leukocytes, once in the tissue, can migrate to the site of inflammation through the gradient of local GAG-bound chemokines. Analogously, endogenous HS-dependent gradients of CCL21 were detected within mouse skin, guiding dendritic cells toward lymphatic vessels (83). These data support the hypothesis that chemokine production at sites of inflammation results in the generation of GAG-mediated chemokine gradients and chemokine presentation by GAGs on the endothelial cell surface, thereby preventing their diffusion and degradation and retaining a high local concentration of the chemokines (52). Finally, blood vessels pattern HS gradients between the apical and basolateral axis (84). Resting and inflamed postcapillary skin venules, as well as high endothelial venules of lymph nodes, show higher HS densities in the basal lamina. Furthermore, the luminal glycocalyx of skin vessels and microvascular dermal cells contained much lower HS densities than their basolateral ECM. Noteworthy, progressive skin inflammation by intradermal injections of complete Freund's adjuvant resulted in massive ECM deposition and in further enrichment of the HS content nearby inflamed vessels. Recently, silencing of exostosin-1, a key enzyme in the biosynthesis of HS, was shown to reduce the directional guidance of neutrophils across inflamed endothelial barriers (85). This again suggesting an important role for basolateral HS. Strikingly, however, effector T cell transendothelial migration is not altered upon silencing of exostosin-1, suggesting that chemotactic signals from intra-endothelial chemokine stores are sufficient to induce the migration of effector T cells.

### Binding to Glycosaminoglycans Is Indispensable for Chemokine Activity *in vivo*

The binding of chemokines to GAGs and oligomerization have been proven to be indispensable for chemokine activity *in vivo* (45, 48, 86, 87). Proudfoot et al. demonstrated that mutations in the GAG-binding sites of CCL2, CCL4 and CCL5 result in abrogated GAG binding and a compromised recruitment of cells *in vivo* when injected intraperitoneally, although receptor binding and *in vitro* chemotactic activity in Boyden chemotaxis chambers were seldom affected. Even at a dose 10,000-fold higher than the active dose of the wild-type chemokines, the mutants with reduced affinity for GAGs showed no activity *in vivo*. Noteworthy, the losses in potency *in vitro* can be attributed to the small losses of receptor affinity and to the impaired interaction with GAGs on the recruited cells, because GAGs can enhance the localization of chemokines to these cells *in vitro* (88). This also indicates that in general chemokines do not need to be immobilized on GAGs to induce chemotaxis *in vitro*. However, recently cis presentation of CXCL4 on GAGs, expressed on leukocytes, was reported to affect *in vitro* cell migration (89).

In addition, inactivation of bifunctional HS N-deacetylase sulphotransferase (NDST-1) in endothelial cells, which is required for sulphation of HS chains, results in impaired neutrophil infiltration in various inflammation models, although these mutant mice develop normally (53). The neutrophil adhesion and migration were reduced because of impaired chemokine transcytosis across the endothelium and reduced chemokine presentation on the endothelial surface. In addition, neutrophil infiltration was decreased to a certain extent due to altered rolling velocity and weaker binding of L-selectin to endothelial cells. In summary, endothelial HS has an important function during inflammation: acting as a ligand for L-selectin during neutrophil rolling, playing a role in chemokine transcytosis and being responsible for the binding and presentation of chemokines at the luminal surface of the endothelium.

### Selectivity and Specificity in Chemokine-Glycosaminoglycan Interactions

Most chemokines are highly basic proteins and therefore it was stated that chemokine-GAG binding largely depends on non-specific electrostatic interactions. However, a certain degree of specificity mediated by van der Waals and hydrogen bonds has been ascribed to this interaction. This was exemplified by binding of the acidic chemokines CCL3 and CCL4 to GAGs (8, 45). In addition, the electrostatic interactions do not necessarily reflect the binding capacity of chemokines to heparin. Although CXCL11 and CXCL12 bound with higher affinity to a non-specific cation exchange resin than CCL5, CCL5 bound stronger to the heparin Sepharose column (9). Before, Kuschert et al. described that GAGs interact with chemokines in a selective manner, providing evidence for GAG sequence specificity. At first, chemokines were shown to exhibit a wide variation in the affinity for heparin and endothelial cells, with, for example, higher affinity binding of CCL5 compared to CCL3 (90, 91). Second, chemokines were shown to possess selectivity in the strength of interaction with GAGs, suggesting that chemokines can discriminate between them. For CCL5, the order is heparin, DS, HS, CS, whereas for CXCL8 and CCL2 the order is heparin, HS, CS and DS. Further, CXCL8 and CXCL1 were shown to bind preferentially to a subset of heparin molecules, whereas CXCL4 and CXCL7 did not show this preference (92). It is important to realize that almost all studies rely on the use of natural GAGs, which are heterogeneous in length and carboxylation and sulphation patterns. Detailed knowledge on interaction of proteins with specific GAG structures depends on the availability of well-described, homogeneous GAG structures. However, chemical synthesis of such specific GAG structures is far more complex than the synthesis of oligonucleotides or peptides.

Several groups have determined the GAG-binding sites in chemokines by using mutagenesis studies, nuclear magnetic resonance (NMR) spectroscopy and mass spectrometry. These studies revealed that typically basic amino acids (Arg, Lys, and His) are involved in GAG binding and that the main GAG-binding motifs on chemokines frequently take the form BBXB or BBBXXBX, in which B and X represent a basic and any amino acid, respectively (93). First, it was stated that the GAG-binding domains are located at a site distant from the specific receptor-binding domain, often within the COOH-terminus of the chemokines. However, the GAG-binding domains were located sometimes in the 40 s loop or in the 20 s loop of chemokines. On the other hand, the GAG-binding motif of CXCL10 is a more widely distributed non-BBXB pattern. Thus, for some chemokines the GAG-binding site is not restricted to the COOH-terminus and has an overlap with receptor-binding sites. Therefore, the question whether chemokines simultaneously bind to GAGs on the endothelium and to their receptor on leukocytes remains unanswered and may be chemokine-dependent. Since chemokines show distinctly different GAG-binding epitopes, these data provide a strong indication for specificity of chemokine-GAG binding.

In addition to the GAG-binding motifs of chemokines, specific chemokine-binding epitopes on GAGs have been identified. Although, for example, N-sulphated groups on HS were not necessary, 2-O-sulphated groups on the iduronic acid units were required for the formation of a GAG-dependent CXCL4 tetramer (94). In addition, a binding site for CXCL8 and CCL3 was identified on HS (95, 96).

### Oligomerization of Chemokines by Glycosaminoglycan Binding

Many chemokines form dimers or higher-order oligomers, thereby adding more complexity to the structural biology of the chemokine system (97). In addition, CXCL12 dimerization was reported to depend on the presence of heparin (98). CXC chemokines dimerize through the interaction of residues in their β1-strands, thereby forming a six-stranded β-sheet structure topped by two α-helices. Importantly, this dimer structure leaves the NH_2_-terminus, N-loop and β3-strand exposed on the surface of the dimer. In this way, CXC chemokine dimers still bind and activate chemokine receptors. In contrast, many CC chemokines dimerize into elongated structures by the formation of an antiparallel β-sheet between the NH_2_-terminal regions. Therefore, it was stated that CC chemokine dimers are inactive. In addition to dimers, several chemokines form higher-order oligomers. For example, CXCL4 and CCL3 form tetramers and polymers, respectively (97).

As discussed before, chemokine-GAG binding is important for the localization and the presentation of chemokines on cell surfaces as haptotactic gradients. Moreover, many chemokines oligomerize on GAGs and are stabilized by GAG binding. This chemokine oligomerization and stabilization is essential for chemokine activity *in vivo* (45, 86, 87, 91, 99). For example, monomeric P8A-CCL2 was incapable of recruiting leukocytes in two *in vivo* models of inflammation. Surprisingly, *in vitro*, the monomeric variants are fully active. Also in other studies, monomeric forms of CXCL8, CCL5, CCL4 and the non-oligomerizing chemokine CCL7 have been shown to bind their receptor and to induce chemotaxis *in vitro* (100–103). Therefore, it can be stated that the monomeric form is sufficient for receptor binding and induction of the directed migration of cells. However, some steps in the process of *in vivo* migration may involve oligomers. Sometimes the monomeric and dimeric forms of the chemokine show different receptor binding and GAG interactions. Both interactions are essential for *in vivo* activity, as exemplified by CXCL8 (104). Moreover, the steepness of the chemokine gradient determined by reversible oligomerization is an important factor in the chemotactic response (104, 105).

It was even stated that oligomerization of chemokines increases their affinity for GAGs by providing a more extensive binding surface. In the presence of GAGs, CCL2 formed a tetramer, whereas normally only a dimer is formed (106). In contrast, the CXCL4 tetramer is stable in the absence of GAGs. Dyer et al. showed that oligomerization-deficient mutants of CCL5 and CXCL4 have reduced affinity for heparin, HS and CS compared with their wild-type counterparts (99). In addition, oligomerization may be required for chemokines to simultaneously bind the receptor and the GAG. Certainly, when the chemokine has overlapping GAG- and receptor-binding sites. Alternatively, Graham et al. suggested a “chemokine cloud” model in which chemokines are presented as molecules sequestered in “solution” in a hydrated glycocalyx (107).

In summary, chemokine oligomerization may be important for the local concentration of the chemokine, thereby preventing their diffusion and degradation. Indeed, GAGs protected chemokines from degradation. CCL11 binding to heparin protected the chemokine from proteolysis by plasmin, cathepsin G and elastase (55). In addition, heparin and HS specifically prevented the processing of CXCL12 by CD26/dipeptidyl peptidase IV (DPP IV) (54, 56). Since cleavage of chemokines by proteases can affect their activity, this protection can serve as an additional degree of regulation prolonging the duration of the chemokine signal (108).

## The Binding of Chemokines to Glycosaminoglycans

### The Binding of ELR^+^ CXC Chemokines to Glycosaminoglycans

#### Consequences of CXCL1, CXCL2, and CXCL6 Binding to Glycosaminoglycans

Already in the 1980s, CXCL2 was described as a GAG-binding protein secreted by monocytes and macrophages and inducing the migration of polymorphonuclear leukocytes (109, 110). However, only years later Wang et al. described the *in vivo* importance of CXCL2 binding to GAGs (53). In mice with endothelial HS deficiency (Ndst ^−/−^ mice) the migration of neutrophils in response to CXCL1 and CXCL2 was significantly decreased. Moreover, in case of CXCL1, the immobilization on the endothelium was decreased in Ndst^−/−^ mice and binding to CXCR2 was dependent on HSPGs (111). In addition, KSPGs formed a chemokine gradient to mediate infiltration of neutrophils to the cornea through interaction with CXCL1, indicating the importance of these PG/CXCL1 complexes in the inflammatory response in eye inflammation (112, 113). A study using CXCL2 mutants with impaired GAG binding also demonstrated that GAG regulation of chemokine activity is tissue-dependent (114). An overview of the processes that are affected by chemokine-GAG interactions is displayed in Table 1.

**Table 1:** Overview of the processes that are affected by chemokine-GAG interactions.

**Chemokine**	**GAG**	**Affected process**	**References**
CXCL1	Heparin, HS	Stability of CXCL1 homodimer, formation of chemokine gradient for cellular trafficking, neutrophil migration in the lung	(115–118)
	HS	Binding to CXCR2 and neutrophil migration *in vivo*	(53, 111, 119)
	KS	Gradient formation in inflammatory response in the eye	(112, 113)
CXCL2	Heparin	GAG/CXCL2/CXCR2 complex formation	(114)
		Stability of CXCL2 homodimer	(115)
		Neutrophil migration in the lung	(118)
	HS	Neutrophil migration *in vivo* in response to CXCL2	(53, 119)
CXCL4	Heparin, HS, CS	High affinity binding	(92, 94, 120–124)
	Cellular GAGs	Prevention of degradation	(125, 126)
CXCL5	Heparin	Heterodimer formation *in vivo*	(127)
CXCL6	HS	High affinity binding	(119)
CXCL7	Heparin	Heterodimer formation *in vivo*	(128)
CXCL8	Heparin, DS, CS, HA	High affinity binding	(129–132)
	HS	CXCL8-induced formation of reactive oxygen species and *in vitro* chemotaxis of	
		neutrophils	(133, 134)
		Inhibition of elastase release	(135)
		High affinity binding	(129)
		Neutrophil activity *in vivo*, inhibition of elastase release from neutrophils	(135)
	Endothelial GAGs	*In vivo* neutrophil migration, transcytosis	(57)
		Oligomerization	(91)
CXCL9	Heparin, CS, HS	Protection from CD26/DPPIV activity	(54)
	HS	Recruitment of plasmacytoid cells	(136)
	Endothelial GAGs	Recruitment and transendothelial migration of T cells	(137)
CXCL10	Heparin, HS	High affinity binding	(138–140)
		Oligomerization	(141)
		Recruitment of plasmacytoid cells	(136)
		Anti-proliferative effect on endothelial cells	(138)
		Anti-fibrotic effect in lungs	(142, 143)
		Antiviral effect against Dengue virus	(144)
	Endothelial GAGs	Recruitment and transendothelial migration of T cells	(137)
CXCL11	Heparin	Cell migration *in vivo*	(142, 145)
		High affinity binding	(99, 119)
	HS	High affinity binding	(99, 119)
		Recruitment of plasmacytoid cells	(136)
	Endothelial GAGs	Recruitment and transendothelial migration of T cells	(137)
CXCL12	Heparin, HS	High affinity binding	(146–151)
		Oligomerization	(99)
		Protection from CD26/DPPIV activity	(56)
		T cell activation in rheumatoid arthritis synovium	(150, 151)
		Intraperitoneal leukocyte accumulation and angiogenesis	(148)
		Anti-HIV activity	(152)
	Heparin, HA, CS, DS	High affinity binding	(146, 149, 153, 154)
CCL2	Heparin, HS	High affinity binding	(99, 103, 155, 156)
		Oligomerization	(106, 157)
		Heterodimerization	(158)
		*In vivo* cell recruitment	(106)
	Heparin, HS, HA, CS, cellular GAGs	High affinity binding	(90, 91, 159)
CCL3	Heparin, HS, DS, CS	High affinity binding and oligomerization	(88, 90, 91, 96, 160, 161)
CCL4	Heparin, HS, DS, CS	High affinity binding and oligomerization	(88, 90, 162–164) (91, 165)
CCL5	Heparin, HS	High affinity binding	(88)
		Oligomerization	(166, 167)
		*In vivo* biological function	(168–170)
		Firm adhesion of leukocytes to endothelial cells, transendothelial migration of macrophages *in vitro*	(168, 171)
		CCL5-dependent apoptosis in T cells	(172)
CCL7	Heparin, HS	High affinity binding	(103, 155, 156, 173)
		Recruitment of leukocytes *in vivo*	(49, 106, 157)
		Heterodimerization	(158)
CCL8	Heparin	Oligomerization	(106, 157)
CCL13	Heparin	High affinity binding	(173)
		Heterodimerization	(158)

*GAG, glycosaminoglycan; HS, Heparan sulfate; KS, keratan sulfate; CS, chondroitin sulfate; DS, dermatan sulfate; HA, hyaluronic acid; DPPIV, dipeptidyl peptidase IV; HIV, human immunodeficiency virus*.

Rajasekaran et al. identified important GAG-binding residues in CXCL2, e.g., Asp19, Lys21, Lys61, Lys65, and Lys69 by NMR spectroscopy (114). Heparin binding enhanced the stability of the CXCL1 and CXCL2 homodimers (115). This enhanced stability upon interaction with GAGs is suggested to increase the lifetime of chemokines, thereby regulating the *in vivo* neutrophil recruitment. The GAG interactions with CXCL2 did not interfere with receptor binding and promoted formation of the GAG/CXCL2/CXCR2 complex. In contrast, two GAG-binding epitopes were identified in CXCL1 as an α-domain, consisting of residues in the N-loop and in the COOH-terminal helix, and a β-domain, consisting of residues in the NH_2_-terminus, 40s loop and the third β-strand indicating an extensive overlap of the GAG-binding and receptor-binding domains (116). CXCL1 mutants with impaired GAG-binding affinity clearly showed reduced neutrophil recruitment to the peritoneum (117). Recently, K_D_-values below 100 nM for CXCL1, CXCL2, and CXCL6 on HS were determined by surface plasmon resonance (SPR) analysis (119). Finally, it was shown that CXCL10 and a COOH-terminal GAG-binding peptide of CXCL9 were able to compete with CXCL1 for GAG binding (111, 174).

A study performed by Tanino et al. showed clear differences in GAG binding between CXCL1 and CXCL2 (118). Due to more rapid association and dissociation of murine CXCL1 from immobilized heparin, CXCL1 was more effective in the recruitment of neutrophils compared to CXCL2. This suggests that chemokines, such as CXCL2, form gradients relatively slowly compared to chemokines that interact with rapid kinetics to GAGs. Thus, different types of chemokine gradients may be formed during an inflammatory response suggesting a new model, whereby GAGs control the spatiotemporal formation of chemokine gradients and neutrophil migration in tissue (118).

#### Consequences of CXCL5 and CXCL7 Binding to Glycosaminoglycans

More than 20 years ago CXCL5 and CXCL7 were purified from epithelial cells and platelets, respectively, using heparin Sepharose chromatography (175–177). Only recently, the basic residues important for GAG binding were identified by NMR spectroscopy (127, 128). Those studies demonstrated that several residues involved in GAG binding are also involved in receptor binding, indicating that the GAG-bound monomer cannot activate its receptor. For CXCL5, the dimer is the high-affinity binding ligand with lysine residues from the N-loop, 40 s turn, β3-strand and COOH-terminal helix being important for GAG binding. In addition, it is known that CXCL7 forms heterodimers with other chemokines, e.g., CXCL1. This CXCL1/CXCL7 heterodimer interacts differently with GAGs compared to the CXCL7 monomer and the GAG-bound heterodimer cannot interact with the receptor (178). These data suggest that GAG interactions play a prominent role in determining heterodimer function *in vivo*.

#### Consequences of CXCL8 Binding to Glycosaminoglycans

CXCL8 is a pro-inflammatory member of the CXC chemokine family attracting polymorphonuclear neutrophils. This chemokine is released at sites of inflammation by cytokine-activated endothelial cells. CXCL8 triggers neutrophils via its specific GPCRs, CXCR1, and CXCR2. In addition, CXCL8 binds to GAGs on the endothelium (129). In 1993, Webb et al. described that progressive COOH-terminal truncation of CXCL8 decreased the affinity for heparin Sepharose (135). In addition, Nordsieck et al. showed that COOH-terminal truncation of this chemokine resulted in an affinity loss of CXCL8 for GAGs due to an alteration of its GAG-binding site (179). Moreover, addition of HS to CXCL8 in a Boyden chemotaxis assay increased the neutrophil chemotactic activity *in vitro*. In contrast, co-incubation of CXCL8 with heparin or dextran sulfate decreased the chemotaxis of neutrophils (133, 134). Also *in vivo* the effects of GAG binding to CXCL8 were not that clear. First, the COOH-terminus was confirmed to be important for GAG binding, transcytosis and the *in vivo* activity of CXCL8 (57). However, several CXCL8 mutants with impaired GAG binding showed higher chemoattractant activity for neutrophils when instilled into the lungs of mice (118).

CXCL8 also bound GAGs on endothelial cells and HS beads with affinities in the micromolar range (90). However, this GAG binding was inhibited by the addition of soluble GAGs. Surprisingly, different GAGs competed differentially with binding of the chemokine to immobilized GAGs, suggesting selectivity. Moreover, the presence of soluble GAGs reduced the receptor binding and the resulting calcium flux. Interestingly, GAGs could alter neutrophil responses, inhibiting the release of elastase from stimulated neutrophils and enhancing the CXCL8-induced formation of reactive oxygen species (ROS) in neutrophils (133, 135).

**Figure 2:**
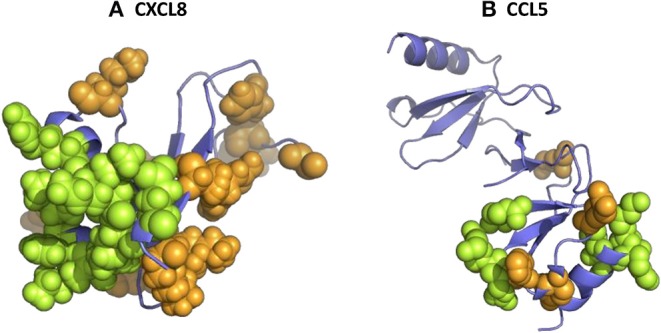
The 3D structure of human CXCL8 and CCL5 and their glycosaminoglycan (GAG)-binding amino acids. 3D models of human CXCL8 **(A)** and CCL5 **(B)** were drawn from PDB accession codes 4XDX and 5COY, respectively, to visualize the location of the amino acids which were shown to be important for GAG binding (green). In addition, other basic amino acids are visualized in orange.

More recently, another study with GAG hexasaccharides confirmed the micromolar affinities (130). Although high-affinity binding for both chondroitin-6-sulfate and heparin was determined, the binding constants for chondroitin-4-sulfate, DS and HA were considerably lower. These data indicate that the 6-O-sulfate groups in chondroitin-6-sulfate and heparin/HS are important for the interaction with CXCL8. The binding of CXCL8 to GAGs is driven by strong ionic interactions between the sulfate groups of the carbohydrates and the basic residues of the protein. In particular, the basic residues His23 and Lys25 in the proximal loop and Arg65, Lys69, Lys72, and Arg73 located in the COOH-terminal α-helix of CXCL8(1–77) were binding anchors for the anionic GAGs (Figure 2A) (180, 181). More recently, the importance of Lys25, Lys69, Lys72, and Glu75 for GAG binding was confirmed and evidenced (131, 132). Interestingly, using affinity co-electrophoresis, it was suggested that conserved glucuronic acid residues in the putative GAG-binding domain of CXCL8 confer GAG selectivity in chemokines. CXCL8 preferentially bound a subfraction of heparin, which was not preferentially bound by CXCL4 (92). Therefore, it was suggested that GAGs are able to determine the specificity of leukocyte recruitment *in vivo*. In addition, it was proven that the length of GAGs plays an important role for CXCL8 binding (90).

Hoogewerf et al. also revealed the importance of GAGs for the oligomerization of chemokines (91). However, the length of GAGs involved in the experimental set-up gave rise to different results. GAG oligosaccharides with chain lengths of up to 16 monosaccharide units showed higher affinity to monomeric CXCL8. In contrast, GAG 22- to 24-mers interacted well with dimeric CXCL8, which can be explained by conformational differences. Longer GAGs contain a more flexible and less sulphated linker domain which connects two terminal, fully sulphated NS-domains thereby forming a horseshoe-like conformation (95, 182). Joseph et al. investigated the structural basis underlying binding of CXCL8 monomers and dimers to GAGs. The CXCL8 dimer was shown to be the high-affinity GAG ligand. In addition, evidence was provided that the binding interface is structurally plastic, thereby mediating a multiplicity of CXCL8-GAG binding interactions. The amino acid residues involved in binding to the GAG comprise a set of core residues that function as the major recognition/binding site and a set of residues in the periphery of the core residues that define the binding geometries of the interaction (183).

### The Binding of ELR^−^ CXC Chemokines to Glycosaminoglycans

#### Consequences of CXCL4 and CXCL4L1 Binding to Glycosaminoglycans

One of the first properties assigned to CXCL4 was its strong affinity for GAGs. In 1976, Levine et al. introduced a purification method to isolate human CXCL4 from activated platelets, namely heparin Sepharose affinity chromatography (120, 184). In addition, CXCL4 showed high affinity for other GAGs, including HS and CS (121). CXCL4 was secreted as a tetramer in a complex with two molecules of CSPGs. In contrast, only one HS bound to the CXCL4 tetramer (94). Using SPR analysis, K_D_-values of CXCL4 for GAGs in the nanomolar range were determined (92, 122–124, 185). Interestingly, CXCL4L1, which only differs from CXCL4 in three COOH-terminal amino acids, had significantly reduced GAG-binding properties (125, 186, 187). Moreover, CXCL4L1 lost its ability to bind to CS. Therefore, it can be stated that CXCL4L1 is less tightly associated to the cell surface than CXCL4 and diffuses much more efficiently after secretion. In contrast, CXCL4 was released by activated platelets in the circulation and subsequently bound to the cell surface leading to rapid clearance from the blood and prevention of its degradation (125, 126). Treatment with heparin resulted in the release of CXCL4 into the circulation (188).

First, a cluster of four lysine residues in the COOH-terminal part of CXCL4 was believed to be critical for GAG binding (94, 120). An analog of CXCL4, with mutations in the four lysines at the COOH-terminus, showed complete loss of heparin binding but retained the ability to suppress the growth of tumors in mice (125, 189). However, other amino acids such as Arg22, His23, Arg24, Tyr25, Lys46, and Arg49 were also involved in the binding to GAGs (123). More recently, Leu67 was shown to be critical for the GAG affinity and Pro58 for binding to CS. In addition, an oligomerization-deficient mutant of CXCL4 had reduced affinity for GAGs compared to wild-type CXCL4 (99).

Recently, a multifunctional protein, TNF-stimulated gene (TSG)-6, was shown to interact with CXCL4 thereby blocking its interactions with GAGs and modulating the inflammatory response (190). In addition, TSG-6 bound GAGs directly, thereby limiting the available GAGs for chemokine interactions.

#### Consequences of CXCL9, CXCL10, and CXCL11 Binding to Glycosaminoglycans

The three CXCR3 ligands, CXCL9, CXCL10, and CXCL11, attract activated T lymphocytes and NK cells and interact with GAGs to conduct their *in vivo* function. Luster et al. first described the binding of CXCL10 or interferon-gamma-inducible protein-10 to cell surface HSPGs on a variety of cells including endothelial, epithelial and haematopoietic cells (138). Originally, it was stated that the chemokine-GAG binding and receptor-binding domain are distinct. However, for CXCL10, experimental evidence exists that the heparin- and CXCR3-binding sites are partially overlapping (139). Mutations of residues 20–24 and 46–47 caused both reduced heparin binding and reduced CXCR3 binding and signaling. For CXCL11, it was described that the COOH-terminus plays an important role in GAG binding since cleavage of CXCL11(5–73) to CXCL11(5–58) by matrix metalloproteinases (MMP) results in loss of heparin binding (145). Indeed, mutations of Lys17 and of basic residues in the COOH-terminal loop, namely ^57^KSKQAR^62^, impaired heparin binding without altering the affinity for CXCR3, indicating distinct heparin- and CXCR3-binding sites (142). However, the mutant was unable to induce cell migration *in vivo*. Interestingly, CXCL11(5–73) was a CXCR3 antagonist with enhanced affinity for heparin (145). In addition, citrullination, the deamination of Arg at position 5 into citrulline, decreased the heparin-binding properties of both CXCL10 and CXCL11 (191). Recently, SPR analysis revealed that murine CXCL10 has a higher affinity for HS than murine CXCL11 with affinities in the nanomolar range (119). As an important note, another study reported different affinities of CXCL11 for heparin and HS (below 10 nM) and revealed an important role for O-sulphation since the affinity of CXCL11 for 2-O-desulphated heparin was reduced (99). Since CXCL9 competed with CXCL8 for binding to heparin, the former chemokine was shown to bind GAGs. More recently, it was shown that GAGs protect CXCR3 ligands against processing by CD26/DPP IV and interfere with receptor signaling (54).

The recruitment of plasmacytoid dendritic cells is mediated by CXCR3, which encounters its ligands (CXCL9, CXCL10, and CXCL11) immobilized by HS (136). Furthermore, the arterial recruitment and the transendothelial migration of T cells was inhibited by soluble heparin which competes with CXCL9, CXCL10, and CXCL11 for binding to endothelial GAGs (137). This phenomenon may contribute to the therapeutic effect of heparin in inflammatory arterial diseases and supports the use of non-anticoagulant heparin derivatives as novel anti-inflammatory therapy. In addition, there is experimental evidence that GAGs not only directly regulate CXCR3 ligand function by chemokine binding. HA fragments, derived from the ECM, were demonstrated to synergize with IFN-γ, leading to enhanced CXCL9 expression in macrophages via NFκB (192). In addition, HA fragments induced the production of CXCL8 and CXCL10 in primary airway epithelial cells in a mitogen-activated protein (MAP) kinase or NFκB-dependent pathway, respectively (193). Noteworthy, this induction was specific for low-molecular-weight HA fragments. In contrast, heparin inhibited the stimulatory effect of IFN-γ on the production of CXCL9 and CXCL10 by human breast cancer cells by inhibiting cellular IFN-γ binding and modulating the IFN-γ-induced signal transducer and activator of transcription 1 (STAT1) phosphorylation (194). CXCL10 is also active on other cell types, such as endothelial cells and fibroblasts. Campanella et al. demonstrated that CXCL10 had anti-proliferative effects on endothelial cells independent of CXCR3 (195). Furthermore, it was suggested that this anti-proliferative effect and the angiostatic properties on endothelial cells are mediated by its specific HS binding site (138). However, there is experimental evidence that the angiostatic effect of CXCL10 in human melanoma was not dependent on GAGs, but was mediated by CXCR3 (196). It was even stated that Arg22 is essential for both CXCR3 binding and angiostasis. In addition, the anti-fibrotic effects of CXCL10 in lungs of mice, in the infarcted myocardium and in cardiac fibroblasts were independent of CXCR3 and required GAG binding (143, 197). Interestingly, the heparin-binding domains of CXCL10 and CXCL11, but not CXCL9, were also involved in binding to the ECM proteins fibrinogen and fibronectin (198). Moreover, fibronectin and CXCL11 synergized in keratinocyte migration and in wound healing *in vivo*, suggesting that interactions between chemokines and the ECM are not restricted to GAG binding.

Also, for CXCL10, oligomerization induced by GAG binding was required for its presentation on endothelial cells and *in vivo* activity (45, 86, 141). Furthermore, oligomerization of chemokines enhanced their affinity for GAGs and affected their ability to be presented by HS (141). In addition, chemokines rigidified and cross-linked HS, thereby changing the mobility of HS. Therefore, it was suggested that chemokine-GAG interactions may promote receptor-independent events such as the rearrangement of the endothelial ECM and signaling through PGs. CXCL11 also displays conformational heterogeneity, explaining the multiple affinity states of CXCL11 for CXCR3 and heparin (142). In addition, interaction of the anti-inflammatory protein TSG-6 and CXCL11 through their GAG-binding epitopes was demonstrated (190).

Further, CXCL10 exerted part of its antiviral properties against dengue virus (DENV) through competition with viral binding to cell surface HS (144). Indeed, DENV rapidly induces the expression of CXCL10 in the liver. Along this line, a COOH-terminal GAG-binding CXCL9 fragment inhibited infection of cells with DENV serotype 2, herpes simplex virus-1 and respiratory syncytial virus. The CXCL9-derived peptide inhibited binding of the DENV envelope protein domain II to heparin (199). In this way, these chemokines play another important role in the host defense against viral infection.

#### Consequences of the Interaction of CXCL12 Proteins With Glycosaminoglycans

CXCL12, also known as stromal cell-derived factor-1 (SDF-1), is constitutively expressed within tissues during organogenesis and adult life orchestrating a lot of functions and it is involved in many pathological mechanisms (200). These physiopathological effects are mediated by CXCR4, to which the chemokine binds and triggers cell signaling. In addition, CXCL12 bound to several cell types in a GAG-dependent manner (146–151). For example, CXCL12 bound to PGs on BM endothelial cells, thereby presenting it to haematopoietic progenitor cells (147). In addition, CXCL12 and CXCL12γ were displayed on HSPGs by endothelial cells in rheumatoid arthritis (RA) synovium (150, 151). Furthermore, CXCL12/GAG interaction was mediated by inflammatory cytokines. In all of the above cases, treatment of the tissue with GAG-degrading enzymes or with sodium chlorate reduced or abrogated the binding of the chemokine. The binding of CXCL12 was diminished on GAG-deficient cells as well.

**Figure 3:**
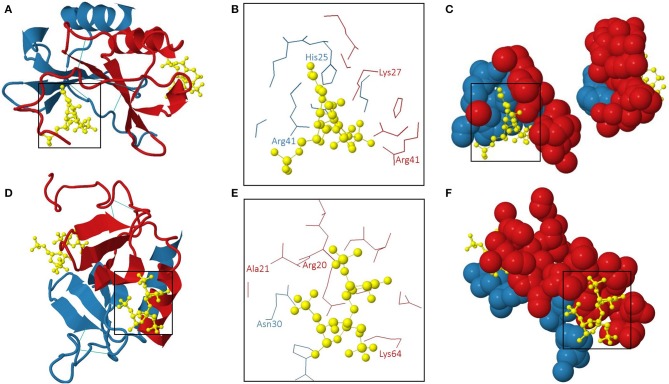
The 3D structure of human CXCL12 dimer: heparin disaccharide complex (202). The 3D model (PDB accession code 2NWG) of the interaction of a human CXCL12 dimer with two heparin disaccharide molecules is shown from two different perspectives in **(A–C)** and **(D–F)**, respectively. **(A,D)**: overview; **(B,E)**: amino acids interacting with heparin disaccharide in the two binding pockets are indicated; **(C,F)**: 3D representation of the individual heparin disaccharide molecules in their binding pockets. The subunits of the CXCL12 dimer are displayed in red (subunit 1) and blue (subunit 2). The heparin disaccharide molecules and disulphide bridges are shown in yellow and light blue, respectively.

Amara et al. demonstrated that CXCL12α binds to heparin with high affinity (K_D_ 38.4 nM) through the first β-strand of the chemokine (146). Indeed, substitution of three basic amino acids in this β-strand, namely Lys24, His25, and Lys27, with Ser impaired the interaction with sensorchip-immobilized heparin. In addition to this typical heparin-binding consensus sequence BBXB, Arg41, and Lys43 played a role in binding of a polysaccharide fragment consisting of 13 monosaccharide units (153). Panitz et al. confirmed the distinct GAG interaction sites of CXCL12 by NMR spectroscopy and molecular modeling (154). Noteworthy, the GAG-binding domains and the receptor-binding sites of CXCL12 were spatially distant (201). Murphy et al. generated an x-ray structure of human CXCL12 in complex with unsaturated heparin disaccharides. Moreover, the specific molecular interactions between the chemokine and heparin were defined (202). The 3D structure of this human CXCL12: heparin disaccharide complex (PDB accession code 2NWG) is shown in Figure 3. Two interaction sites for heparin disaccharide molecules are displayed on a CXCL12 dimer configuration. One heparin disaccharide binds to the dimer interface and forms hydrogen bonds with His25 of subunit 2, Lys27 of subunit 1 and Arg41 of both CXCL12 subunits (Figures 3A–C). The second disaccharide binds to the NH_2_-terminal loop and the α-helix and interacts with Arg20, Ala21, and Lys64 of subunit 1 and Asn30 of subunit 2 of the CXCL12 dimer (Figures 3D–F). His25 and Lys27 belong to a BBXB GAG-binding motif (Lys24 – Lys27) (202). On the contrary, the other aforementioned amino acid residues are not part of GAG-binding motifs, thereby emphasizing the importance of the 3D structural arrangement of positively charged amino acids for the ability to bind GAGs.

In contrast to CXCL12α, the splicing variant CXCL12γ has an extremely long and basic COOH-terminal extension, which contains as much as 18 basic residues, of which 9 being clustered into three putative BBXB HS-binding domains. As expected, CXCL12γ showed much higher affinity for GAGs compared to CXCL12α and CXCL12β (149). Moreover, the unstructured cationic domain of CXCL12γ extended the range of GAGs to which it can bind. The higher affinity of CXCL12γ for GAGs compared to CXCL12α was also shown by enhanced binding to cell surface-expressed HS. In addition, COOH-terminal fragments of CXCL12γ inhibited infection of cells with DENV serotype 2, herpes simplex virus-1 and respiratory syncytial virus (199). In addition, mutant chemokines were developed to evaluate the contribution of the COOH-terminal domain and the core region of CXCL12 in GAG binding. Mutation of the BBXB motif in the core region of both CXCL12β and CXCL12γ resulted in impaired GAG binding. However, mutations of the COOH-terminal domain only increased the off-rate, suggesting that this COOH-terminal domain is necessary for the stability of the chemokine-GAG complex. Although CXCL12γ showed reduced affinity for CXCR4, the sustained binding of this isoform to HS enabled it to promote *in vivo* intraperitoneal leukocyte accumulation and angiogenesis in matrigel with much higher efficiency than CXCL12α (148). This suggests that the γ isoform might exist predominantly in a GAG-bound form within tissues to either stabilize or protect the chemokine from proteolytic cleavage events that directly affect its activity and/or to immobilize CXCL12γ to allow continued and localized stimulation of cells. Indeed, binding of CXCL12α to heparin or HS prevented the proteolytic processing of CXCL12α by CD26/DPP IV (56). More recently, it was described that CXCL12γ interacts with high affinity with sulphotyrosines in the NH_2_-terminal region of CXCR4 resulting in a non-productive binding and reduced signaling and chemotactic activity. However, HS prevented the interaction between CXCL12γ and CXCR4 sulphotyrosines, thereby functionally presenting the chemokine to its receptor such that its activity was similar to that of CXCL12α (203).

As mentioned before, GAG binding is necessary for chemokine function and chemokine oligomerization contributes to the affinity of chemokine-GAG interactions. Recently, a disulphide-locked dimer of CXCL12 showed an increase in affinity for GAGs compared to wild-type CXCL12, which exists as an equilibrium mixture of monomers and dimers (99).

Further, mutant CXCL12α with impaired GAG-binding capacity was not able to prevent the fusion of human immunodeficiency virus (HIV) X4 isolates in leukocytes in the same degree as wild-type CXCL12α (152). Again, the enzymatic removal of cell surface HS diminished the HIV-inhibitory capacity of the chemokine. Also the anti-inflammatory protein TSG-6 interacted with CXCL12α through their GAG-binding epitopes, resulting in a decreased presentation on the endothelial surface (190).

#### Consequences of CXCL14 Binding to Glycosaminoglycans

Penk et al. explored the interaction between CXCL14 and various GAGs by using NMR spectroscopy, molecular modeling, heparin affinity chromatography and mutagenesis. They detected distinct GAG-binding modes dependent on the type of GAG that was used. Accordingly, the binding pose for heparin was suggested to be different from the binding poses of HA, CS-A/C, -D and DS. Moreover, it was proposed that different GAG sulphation patterns might confer specificity to the interaction (204).

### The Binding of CC Chemokines to Glycosaminoglycans

#### Consequences of CCL2, CCL7, CCL8, and CCL13 Binding to Glycosaminoglycans

Hoogewerf et al. and Kuschert et al. described GAG-dependent binding of chemokines, including CCL2, to endothelial and CHO cells (90, 91). Amino acids Lys58 and His66 in the COOH-terminal α-helix of CCL2 were essential for GAG binding (159). However, these were less important than the amino acids Arg18, Lys19, Arg24, and Arg49 (45, 106). The [^18^AA^19^]-CCL2 mutant and the monomeric P8A-CCL2 mutant showed reduced GAG affinity and *in vivo* cell recruitment, although they retained chemotactic activity *in vitro*. Thus, the quaternary structure of chemokines and their interaction with GAGs may contribute to the recruitment of leukocytes beyond migration patterns defined by interactions with chemokine receptors. In addition, CCL7 and CCL13 bound to heparin with comparable affinity (173). A non-GAG-binding CCL7 mutant showed reduced recruitment of leukocytes *in vivo* indicating the importance of a BXBXXB GAG-binding motif (49). Noteworthy, the binding of monocyte attractants CCL2, CCL7, and CCL8 to GAGs was dependent on the position of sulphation, and acetylation (155, 156). More recently, SPR analysis showed high GAG affinity for CCL2 and CCL7 with K_D_-values below 120 nM for heparin or HS (103). In accordance to previously obtained data, the P8A mutant of CCL2 showed reduced GAG-binding affinity to heparin, HS and 2-O-desulphated heparin (99). Mutagenesis studies revealed multiple GAG-binding sites in CCL7, enabling it to function as a non-oligomerizing chemokine (103). Noteworthy, the chemokine receptor CCR2 competed with GAGs for CCL7 binding (205). On the opposite, there exists experimental evidence reporting no interaction between GAGs and CCL2 (119, 206). For example, under physiological salt conditions, no binding of CCL2 to mast cells and the ECM in RA synovium was detected. Noteworthy, the use of GAGs with varying length or pattern of sulphation in the binding assays can result in different binding constants.

CCL2 and CCL8 oligomerized in solution and more profoundly in the presence of GAGs (106, 157). For example, CCL2 dimers and tetramers were formed in the presence of octasaccharides. However, without GAGs, both monomers and dimers of CCL2 and CCL8 were detected. In contrast, CCL13 oligomerized in the presence or absence of GAGs. In addition, CCR2 ligands formed heterodimers, a process which is partially regulated by GAG binding (158). For example, CCL2 formed heterodimers with CCL8, CCL11 and CCL13 and CCL8 heterodimerizes with CCL13. In the presence of GAGs, also CCL8/CCL11 heterodimers were detected. Interestingly, multimerization of CCL2 was not required for transendothelial migration. However, treatment with heparin resulted in reduced GAG binding and the inhibition of migration across the endothelium (207). As described before, PTM of chemokines is an important mechanism to regulate chemokine function. Although these modifications mostly lead to changes in receptor activity, also GAG-binding affinity can be altered. Recently, nitrated CCL2 with reduced *in vitro* and *in vivo* activity was described (208). This could be partially attributed to reduced GAG binding of the nitrated chemokine.

#### Consequences of CCL3, CCL4, and CCL5 Binding to Glycosaminoglycans

As mentioned before, a common feature of GAGs is their overall negative charge suggesting an electrostatic interaction with basic proteins, such as chemokines. However, the chemokine-GAG interaction is not merely based on overall electrostatic interactions as exemplified by the fact that CCL3 and CCL4, both acidic chemokines, bind GAGs (90, 91). These chemokines, including CCL5, bound to GAGs on cells and/or HS beads (88). Several highly conserved basic amino acids were identified by *in vitro* mutagenesis to be involved in GAG binding, including a common heparin-binding motif of the form BBXB in the 40 s loop of CCL3, CCL4, and CCL5 (160–163, 168). The amino acids Arg18, Arg46, and Arg48 of CCL3, amino acids Arg18, Lys45, Arg46, and Lys48 of CCL4 and amino acids Arg44, Lys45, and Arg47 of CCL5 were involved in the GAG interaction (Figure 2B). Interestingly, the alteration of acidic residues in CCL3 led to an enhanced heparin-binding affinity (209). For CCL3, also a binding site on HS was characterized as a domain consisting of two highly sulphated regions (NS), 12–14 monosaccharide units long, separated by an N-acetylated region (NA) (96). The NS domains likely interact with the basic amino acids Arg17, Arg45, and Arg47 and may wrap around the CCL3 dimer in a horseshoe shape. Again, the CCL3 dimer showed higher affinity for HS than the monomeric or tetrameric form. NMR spectroscopy identified other residues of CCL4 involved in GAG binding, namely Arg18, Asn23, Val25, Thr44, Lys45, Lys46, and Ser47 (164). In case of CCL5, another GAG-binding domain, namely residues ^55^KKWVR^59^ in the 50 s loop, was described as the low binding affinity site, whereas the basic amino acids ^44^RKNR^47^ in the 40 s loop of CCL5 served as the main GAG-binding domain (169). Indeed, more recently, it was shown that the basic cluster in the 50 s loop is required for the *in vivo* biological function of CCL5 (170). The ^55^AAWVA^59^-CCL5 mutant lost the capacity to mediate firm adhesion of leukocytes to endothelial cells, transendothelial migration of macrophages *in vitro* and recruitment of cells to the peritoneum *in vivo*. Previously, Burns et al. suggested an important role for the amino acids 55–66 in GAG binding, since a monoclonal antibody recognizing this epitope blocked the GAG-dependent antiviral activity of CCL5 (210, 211).

Surprisingly, the non-heparin binding mutants (R46A) of CCL3 and CCL4 still bound to their receptor with similar potency, inducing similar Ca^2+^-signals or T cell chemotactic responses. For CCL4, two residues, namely Lys48 and Arg45, had overlapping functions playing a critical role in both heparin and CCR5 binding (163). In addition, mutant CHO cells transfected with the GPCRs CCR1 or CCR5 with defective GAG expression still bound CCL3, CCL4, and CCL5, but required exposure to higher chemokine concentrations to induce similar Ca^2+^-responses (88). Several studies with mutant CCL5 molecules showed that the interaction between the chemokine and GAGs is not essential for receptor binding, signal transduction and leukocyte migration (168, 171). However, this interaction was required for transendothelial migration, where the development of a chemokine gradient was important. The ^44^AANA^47^-CCL5 mutant displayed reduced GAG-binding affinity, whereas the ^55^AAWVA^59^-mutant retained full binding capacity (168). Mutations in the 40 s loop also abolished binding to tissue sections, and interestingly, so did mutation of the 50 s region (212). Although the ^44^AANA^47^-CCL5 mutant showed reduced CCR1 binding, the high-affinity binding to CCR5 and the ability to induce chemotaxis of freshly isolated monocytes in a Boyden chamber assay were retained (168). Single point mutations in the putative GAG-binding domains resulted in reduced GAG-binding affinity, but similar chemotactic responses *in vitro* (171). However, as discussed before, in more physiologic conditions the decreased binding to extracellular structures led to reduced biological activity.

As discussed before, GAG binding, but also oligomerization may be essential for the *in vivo* activity of specific chemokines. Indeed, CCL4 and CCL5 mutants with impaired GAG binding and monomeric variants were unable to recruit cells when injected into the peritoneal cavity, although they are fully active *in vitro* (45). In addition, the ^44^AANA^47^-CCL5 mutant was unable to form high-order oligomers, to bind to heparin and to recruit cells *in vivo* (166). This mutant also failed to induce apoptosis in T cells (172). In addition, dimeric CCL4 displayed higher affinity for heparin and disaccharide subunits (163, 164). Moreover, the dimerization affinities of CCL4 and CCL5 increased in the presence of a disaccharide (165). However, the BBXB motifs of CCL3, CCL4, and CCL5 are partially buried when they are oligomerized. For the interaction between GAGs and the CCL3 oligomer, residues from two partially buried BBXB motifs together with other residues are involved. For the CCL5 oligomer another fully exposed motif was important for GAG binding (213). Rek et al. described that CCL5 undergoes a conformational change when it binds to HS (167). This change in conformation was suggested to be a prerequisite for oligomerization and optimal GPCR activation *in vivo*. As described before, TSG-6 was able to bind to chemokines through their GAG-binding domains (190). Pre-incubation of endothelial cells with TSG-6 inhibited the presentation of CCL5 on the endothelial surface.

In contrast to all the above, two different studies could not detect binding of CCL3 and CCL4 to GAGs by SPR analysis (99, 119). Others reported that CCL3 and CCL4 did not bind in a GAG-dependent manner to mast cells and to the ECM in the synovium of RA patients (206). In all these studies, CCL5 binding to GAGs was detected and a mutant CCL5, with decreased avidity for heparin, was not able to bind to mast cells or ECM anymore. These discrepancies in the results of these studies could be explained by the rather acidic nature of both CCL3 and CCL4 compared to other chemokines.

#### Consequences of CCL11 Binding to Glycosaminoglycans

Eotaxin- or CCL11-induced calcium signaling, respiratory burst and migration of eosinophils and binding of CCL11 to CCR3 was inhibited by heparin (173). However, heparin did not affect chemotactic responses to C5a. In addition, heparin inhibited CCL11, CCL24, CCL7, CCL13, and CCL5-induced eosinophil stimulation in different degrees, correlating with their relative affinities for heparin. Although HS and DS inhibited the action of CCL11, no effect was observed with CS. On the contrary, Ellyard et al. showed only binding to heparin and not to HS. Moreover, heparin protected CCL11 from proteolysis, thereby potentiating chemotactic activity *in vivo* (55). Recently, a tetrameric form of CCL11 was shown to bind the therapeutic GAG Arixtra (214).

#### Consequences of CCL19 and CCL21 Binding to Glycosaminoglycans

CCL21 was shown to bind to versican, a large CSPG, via its GAGs (215). Although HS supported CCL21-induced Ca^2+^-mobilization, versican and CS B inhibited cellular responses. Moreover, the COOH-terminus of CCL21 was involved in GAG binding and the inhibitory effect of CS B on the CCL21-induced Ca^2+^-influx (216). The COOH-terminal tail of CCL21 reduced its *in vitro* chemotactic potency in a 3D dendritic cell chemotaxis assay but enhanced its efficiency to activate ERK1/2 signaling and β-arrestin recruitment (217). In addition, full-length CCL21 induced integrin-dependent dendritic cell spreading, polarization and haptotactic movement, whereas CCL21 missing the positively charged COOH-terminus induced non-adhesive and integrin-independent directional migration (218). Interestingly, linking the COOH-terminal tail of CCL21 to the related CCR7 ligand CCL19 enhanced its affinity for heparin (219).

In accordance to CCL5 and CCL17, CCL21 also bound to mast cells and the ECM in RA synovium and this chemokine binding was inhibited by high salt concentrations and GAGs (206). Binding of CCL21 to immobilized heparin was greatly diminished upon human endosulphatase-treatment (140). Both CCL21 and CCL19 bound to a hexasaccharide as observed by SPR analysis (220). GAG binding also plays an essential role in chemokine cooperativity (221). In the absence of cooperative chemokines, CCL19 and CCL21 bound to CCR7 or GAGs on the endothelial cell surface. However, in the presence of cooperative chemokines, CCL19 and CCL21 are competed from GAGs, increasing the concentration of chemokine which can interact with their receptor. Finally, TSG-6 binding to CCL19 and CCL21 was described (190). This interaction resulted in inhibition of chemokine binding to heparin and presentation on the endothelium and the inhibition of CCL19- and CCL21-mediated transendothelial migration.

### The Binding of C Chemokines XCL1 and XCL2 to Glycosaminoglycans

The two lymphocyte attractants XCL1 and XCL2 bound GAGs (222, 223). Both convert between a canonical chemokine folded monomer and a unique dimer. Interestingly, the monomer forms were responsible for receptor binding and activation, whereas the dimer forms were involved in GAG binding. Recently, a major GAG-binding site of XCL1 was determined as mutations of the amino acids Arg23 and Arg43 greatly diminished GAG binding (224). Despite their structural similarity, XCL2 displayed a higher affinity for heparin than XCL1. In addition, the XCL1 dimer was responsible for inhibiting HIV-1 activity.

In summary, it can be stated that the interaction between chemokines and GAGs on the cell surface is not essential for GPCR binding and signaling. However, GAG binding enhances the activity of low chemokine concentrations by sequestration of chemokines on the cell surface, inducing polymerization of chemokines and increasing their local concentration. Therefore, cell surface GAGs enhance the effect of chemokines on high-affinity receptors within the local microenvironment.

## Therapeutic Approaches Inhibiting Chemokine-GAG Interactions

During the last two decades, research aiming at interference with chemokine activity mainly focused on the identification of inhibitors of the interaction between chemokines and their cognate GPCRs. This approach resulted in limited success with a number of compounds in clinical trials, but only two small molecule chemokine receptor antagonists on the market (for treatment of HIV and treatment of leukemia) (225–227). Since it is clear that also binding to GAGs is important for chemokine functioning *in vivo*, a few groups are investigating the inhibition of chemokine-GAG interactions (199, 228–233).

### Viral Chemokine-Binding Proteins

The chemokine network exerts an indispensable role in the antiviral immune response. Accordingly, some viruses have developed strategies to modulate chemokine activity, thereby affecting leukocyte migration and aiming at evasion or manipulation of the host immune response. These viral mechanisms are highly sophisticated as they possibly have been selected during evolution over millions of years. Large DNA viruses, poxviruses and herpesviruses in particular, use a substantial part of their genome to neutralize the antiviral activity of the immune system of the host. One of their strategies involves the expression of proteins that have the ability to modulate chemokine activity: viral chemokine homologs, viral chemokine receptor homologs and viral chemokine-binding proteins (vCKBPs). The latter group includes secreted proteins that display no sequence similarity with mammalian proteins. These vCKBPs can interfere with chemokine function via binding to either the GAG-binding epitope of chemokines or the chemokine receptor-binding epitope of chemokines, resulting in disruption of the chemokine gradient or abrogated interaction of the chemokine with its chemokine receptor, respectively (234–237). In contrast to the observed inhibitory activity of vCKBPs on chemokine function, a vCKBP that does not inhibit, but potentiates chemokine activity has been detected in both herpes simplex virus type 1 (HSV-1) and HSV-2 (238). The fact that viruses produce proteins that disrupt the chemokine gradient emphasizes the importance of the chemokine-GAG interaction (234). In this paragraph, we will focus on vCKBPs that inhibit chemokine activity by interfering with the chemokine-GAG interaction.

#### Poxviruses

##### A41

Vaccinia virus (VACV), used as vaccine for the eradication of smallpox caused by the variola virus, produces and secretes a 30 kDa glycoprotein called A41. SPR experiments could identify the CC chemokines CCL21, CCL25, CCL26, and CCL28 as binding partners for A41 (K_D_ values between 10^−7^ and 10^−9^ M). GAGs could disrupt the interaction of A41 with chemokines, indicating that A41 can inhibit binding of a subset of CC chemokines to GAGs via interaction with a site that overlaps with their GAG-binding site. A41 did not affect binding of these chemokines to their chemokine receptors (239).

##### E163

Ectromelia virus (ECTV) is closely related to the variola virus and is the causative agent of mousepox. Accordingly, ECTV infections in mice have been used as a model to study smallpox (240). ECTV encodes a 31 kDa glycoprotein called E163, which is an ortholog of the A41 protein encoded by VACV. Moreover, E163 has been identified as a vCKBP due to its ability to bind a subset of CC and CXC chemokines with high affinity. By using SPR, Ruiz-Argüello et al. demonstrated high-affinity binding (nanomolar range) of this vCKBP to three CXC chemokines (CXCL12α, CXCL12β, CXCL14) and to six CC chemokines (CCL21, CCL24, CCL25, CCL26, CCL27, CCL28). Neither the interaction of chemokines with specific GPCRs, nor leukocyte chemotaxis *in vitro* could be inhibited by E163. More specifically, heparin dose-dependently competed with chemokines for interaction with E163, suggesting that E163 binds to the GAG-binding site of chemokines and not to their receptor-binding domain. This hypothesis was confirmed as chemokines with mutated GAG-binding sites showed abrogated interaction with E163 (241). In addition, this vCKBP includes three GAG-binding motifs and correspondingly bound to a variety of sulphated GAGs. This interaction enables anchorage of E163 to the cell surface, thereby retaining it in the proximity of the infected tissue. Moreover, binding to GAGs might protect this vCKBP from degradation by proteases (241, 242). Binding of E163 to the GAG-binding domain of chemokines already suggested its potential to inhibit the chemokine-GAG interaction. Heidarieh et al. further investigated this hypothesis and by using GAG-binding mutant forms of E163, they showed that E163 interferes with the interaction between chemokines and GAGs on the cell surface. In addition, E163 appears to have the ability to interact simultaneously with chemokines and GAGs (242).

##### M-T7

Myxoma virus is a poxvirus that exclusively infects rabbits and causes myxomatosis. This virus secretes the myxoma virus T7 protein (M-T7) that is a soluble IFN-γ receptor homolog. Correspondingly, M-T7 bound to rabbit IFN-γ and was a potent inhibitor of the biological activity of this cytokine (243–246). Lalani et al. reported that in addition to the latter function, M-T7 interacted with multiple chemokines of the C, CC, and CXC subclasses (mXCL1, hCCL5, hCCL2, hCCL7, hCXCL8, hCXCL4, hCXCL10, hCXCL7, hCXCL1), which could be observed in a gel shift mobility assay. In contrast to the NH_2_-terminal region of CXCL8, the COOH-terminal region of this chemokine appeared to be required for binding to M-T7 since COOH-terminally truncated forms had lost the ability to interact with M-T7. Moreover, heparin competed with M-T7 for binding to CCL5. Accordingly, it was proposed that M-T7 interacts with the GAG-binding domain of multiple chemokines (245).

##### ORFV CKBP

Orf virus (ORFV) is a parapoxvirus that infects sheep, goats, and humans. Among a range of host-modulating proteins, this virus encodes a vCKBP, namely ORFV CKBP. SPR experiments demonstrated high-affinity binding of the ORFV CKBP to the CC chemokines CCL2, CCL3, CCL4, CCL7, CCL11 and to the C chemokine XCL1 (247). In addition, ORFV CKBP bound with high affinity to CXC chemokines (CXCL2 and CXCL4) (248). Upon interaction between ORFV CKBP and a chemokine, the vCKBP masks key amino acid residues of the chemokine receptor-binding domains and the GAG-binding domains in the chemokine. Accordingly, ORFV CKBP had the ability to block chemokine binding and signaling through its cognate chemokine receptor and interfered with the chemokine-GAG interaction (247, 248).

#### Herpesviruses

##### gG

Bryant et al. identified a family of novel vCKBPs, namely glycoprotein G (gG), encoded by alphaherpesviruses including equine herpesvirus 1 (EHV-1), bovine herpesvirus 1 and 5 (BHV-1 and BHV-5) among others. Secreted forms of gG from some alphaherpesviruses are characterized by a broad binding specificity for chemokines. Moreover, gG can inhibit chemokine activity by interfering with the interaction of chemokines with their cognate chemokine receptors and with GAGs. This was exemplified by the gG vCKBP from EHV-1 that showed the ability to disrupt pre-established chemokine-GAG (CXCL1-heparin) interactions. In addition, gG from EHV-1 and BHV-1 blocked the binding of chemokines to GAGs on the cell surface (249).

##### M3

Murine gammaherpesvirus-68 infects murid rodents and its *M3* gene encodes a vCKBP, namely M3 or vCKBP-3 (250, 251). This vCKBP bound to a broad range of chemokines of all four subclasses (C, CC, CXC, and CX3C chemokines). Initially, the ability of this vCKBP to inhibit binding of chemokines to their GPCRs was demonstrated (250, 252). However, further investigations by Webb et al. revealed that M3 inhibited binding to heparin of a variety of chemokines (CXCL1, CXCL8, CXCL10, CCL2, and CCL5). In addition, M3 blocked the interaction of CCL3 and CXCL8 with cell surface GAGs. Moreover, heparin-bound CCL5 and CXCL8 could be displaced from GAGs by M3 (251). Furthermore, the N-loop of chemokines was demonstrated to be required for binding to M3 (250, 252). So, it is thought that M3 binds to chemokines via their N-loop, thereby resembling binding to the chemokine receptor and apparently disrupting binding to heparin (251).

##### R17

R17 is a vCKBP encoded by rodent herpesvirus Peru (RHVP) that is a gammaherpesvirus related to murine gammaherpesvirus-68. This vCKBP bound a range of human and mouse C- and CC chemokines (hCCL2, hCCL3, hCCL5 and mCCL2, mCCL3, mCCL4, mCCL5, mCCL8, mCCL11, mCCL12, mCCL19, mCCL20, mCCL24, and mXCL1) with high affinity in SPR experiments. Moreover, the interaction of R17 with chemokines abrogated chemokine-mediated cell migration and calcium release, suggesting an inhibitory function on chemokine signaling for R17. In addition, binding of R17 to cell surface GAGs has been observed. R17 comprises two BBXB GAG-binding motifs, which are both crucial for GAG binding, as demonstrated by variants of R17 with mutated GAG-binding motifs. Further experiments showed that the interaction of R17 with GAGs relies on determinants that are distinct from those involved in binding to chemokines (253). Additionally, Lubman et al. used an SPR-based competition experiment to demonstrate the ability of R17 to interfere with the chemokine-GAG interaction for chemokines like CCL2 (254).

### Tick Saliva Protein Evasin-3 and Synthetic Variants

Ticks are bloodsucking parasites that, like many pathogens, have developed certain mechanisms to evade the immune response of their host. Tick saliva contains a wide range of immunomodulatory proteins including a class of CKBPs, termed Evasins. These proteins bound and neutralized chemokines, thereby preventing recruitment of cells of the innate immune system and allowing ticks to remain undetected by their host (255). Until now, the class of Evasins comprises three family members: Evasin-1, Evasin-3 and Evasin-4. Since Evasin-1 and Evasin-4 are structurally related, they constitute the subclass C8 fold, whereas Evasin-3 belongs to the subclass C6 fold containing 8 and 6 cysteines, respectively. The C8 fold Evasins bound to CC chemokines: CCL3, CCL4, CCL18 (Evasin-1) and CCL5, CCL11 (Evasin-4). In contrast, the C6 fold Evasin-3 had high affinity for the CXC chemokines CXCL1 and CXCL8 (and their murine related proteins: CXCL1 and CXCL2). Potent anti-inflammatory activity of Evasins has been demonstrated in several *in vivo* animal models of disease (255, 256).

Recently, Denisov et al. explored the three-dimensional structures of Evasin-3 and the CXCL8 – Evasin-3 complex. Evasin-3 bound to the CXCL8 monomer and disrupted the interaction of CXCL8 with GAGs and with its receptor CXCR2. When Met-Evasin-3 (a variant of Evasin-3 with a methionine residue at the NH_2_-terminus) was added to the CXCL8-GAG complex in *in vitro* experiments, GAG binding was abrogated. Evasin-3 disrupted the continuous stretch of positively charged amino acids of the GAG-binding domain of CXCL8, thereby preventing binding of the chemokine to GAGs. Consequently, Evasin-3 competed with GAG binding and replaced the GAGs from the CXCL8-GAG complex. Furthermore, two novel CXCL8-binding truncated Evasin-3 variants: linear tEv3 17-56 and cyclic tcEv3 16-56 dPG were synthesized. These variants demonstrated high proteolytic stability in human plasma and inhibited CXCL8-induced neutrophil migration *in vitro* to a similar extent as native Evasin-3. Both synthetic Evasin-3 variants showed high affinity for CXCL8, although lower than the affinity of native Evasin-3 for CXCL8. The long NH_2_- and COOH-termini of native Evasin-3 apparently affect the internal dynamics of its structure, resulting in lower K_D_ values (256).

Potent *in vivo* anti-inflammatory activity of Evasin-3 has been shown in several animal models. Evasin-3 inhibited CXCL1-induced neutrophil recruitment to the peritoneal as well as the knee cavity. In addition, Evasin-3 treatment in a murine model of antigen-induced arthritis led to an overall reduction of leukocyte recruitment to the joint and periarticular tissues. Especially neutrophil influx in the knee joint was decreased (by 70%). Moreover, treatment diminished inflammatory hypernociception and the local production of TNF-α. Evasin-3 inhibited the adhesion of leukocytes to the synovial endothelium as observed in intravital microscopy experiments. In addition, Evasin-3 reduced lethality in a murine model of intestinal ischemia-reperfusion injury (255). Evasin-3 treatment was evaluated in a murine model of myocardial ischemia-reperfusion injury as well. A single administration of Evasin-3 during myocardial ischemia resulted in reduced infarct size. This beneficial effect could be allocated to the inhibition of neutrophil influx and the decrease in ROS production (257). Another study demonstrated the positive effect of Evasin-3 on atherosclerotic vulnerability for ischemic stroke. In a mouse model of carotid atherosclerosis, treatment with Evasin-3 resulted in decreased intraplaque neutrophilic inflammation and matrix metalloproteinase-9 content. However, in a murine model of ischemic stroke, no poststroke clinical outcomes were ameliorated by treatment with Evasin-3, suggesting that this treatment is not useful to prevent ischemic brain injury (258). In addition, Evasin-3 reduced neutrophilic inflammation in both lung and pancreas in a murine model of acute pancreatitis. Macrophage recruitment to the pancreas was reduced as well. Evasin-3 treatment reduced ROS release in the lung. Furthermore, treatment with Evasin-3 was associated with reduced lung and pancreas apoptosis and pancreas necrosis (259).

### Human Chemokine-Binding Protein TNF-Stimulated Gene-6

TSG-6 is an inflammation-associated protein with tissue-protective and anti-inflammatory characteristics. Its therapeutic effects have been studied in a wide range of disease models. TSG-6 interacted with various ligands including GAGs and its ability to bind to chemokines has been revealed recently. TSG-6 is the first soluble CKBP discovered in mammals (260, 261). This CKBP bound chemokines of the CC and CXC subfamilies (CCL2, CCL5, CCL7, CCL19, CCL21, CCL27, CXCL4, CXCL8, CXCL11, and CXCL12) and associated with their GAG-binding site, interfering with their interaction with GAGs (190, 262).

### Chemokine-Derived GAG-Binding Peptides

GAG-binding peptides can be used as another strategy to target the chemokine-GAG interaction. The design of several GAG-binding peptides derived from different chemokines has been reported. We synthesized a CXCL9-derived GAG-binding peptide, namely CXCL9(74–103). In contrast to most other chemokines, the COOH-terminal region of CXCL9 is exceptionally long, highly positively charged and conserved among species. CXCL9 is a CXCR3 ligand and recruits activated T lymphocytes and NK cells. Moreover, this chemokine has angiostatic properties (16, 231, 263). Remarkably, when natural CXCL9 was purified, the highly charged COOH-terminus (a peptide of up to 30 amino acids) was almost always cleaved from the intact chemokine (231). In a next step, the potential role of this natural COOH-terminal peptide was evaluated. COOH-terminal CXCL9-derived peptides with different length were synthesized. These CXCL9-derived peptides do neither activate, nor recruit leukocytes through CXCR3 (231).

CXCL9(74–103) bound with high affinity to soluble and cellular GAGs. The longest 30 amino acid peptide, CXCL9(74–103), was the most potent competitor and competed with CXCL8, muCXCL1, muCXCL6, CXCL11, CCL2, and CCL3 for binding to GAGs (174, 231, 264). This indicated that CXCL9(74–103) may compete with a wide variety of chemokines that belong to different subclasses. Furthermore, the importance of amino acids 74–78 was emphasized as CXCL9(74–103) was the most potent GAG-binding peptide. Although these amino acids were required, they were not sufficient for GAG binding. CXCL9(74–103) included two typical GAG-binding motifs (BBXB: ^75^KKQK^78^ and BBBXXB: ^85^KKKVLK^90^) (231). A shorter peptide, CXCL9(74–93) showed similar affinity for HS and LMWH in comparison with CXCL9(74–103). However, the affinity of the shorter peptide for binding to CS was lower compared with CXCL9(74–103), indicating that shortening of CXCL9(74–103) resulted in a narrowing of the GAG-binding spectrum (174).

Since CXCL9(74–103) competed with intact chemokines for binding to GAGs *in vitro* and showed abrogated binding to and signaling through CXCR3, it was hypothesized that CXCL9(74–103) would compete with active chemokines for GAG binding *in vivo*, thereby inhibiting leukocyte migration. In addition, as CXCL9(74–103) is derived from a chemokine, it might show specificity for certain GAG sequences expressed on the endothelium in an inflammatory situation (231). The potential anti-inflammatory activity of this peptide was first assessed in two murine acute inflammation models characterized by neutrophil infiltration. The CXCL9(74–103) peptide competed with the most potent human neutrophil-attracting chemokine, i.e., CXCL8 for GAG binding and blocked neutrophil migration in both a gout model and an inflammation model that involved intra-articular injection with CXCL8 (231). Furthermore, treatment with CXCL9(74–103) in a murine model of CXCL8-induced neutrophil recruitment to the peritoneal cavity reduced the potency of CXCL8 to induce neutrophil infiltration. In an intravital microscopy experiment, binding of CXCL9(74–103) to the endothelium was visualized in the murine cremaster muscle model. Administration of CXCL8 resulted in neutrophil recruitment. However, treatment with CXCL9(74–103) led to decreased adherence of neutrophils to the endothelial cells (174). In a murine model of antigen-induced arthritis the peptide reduced the recruitment of leukocytes, especially neutrophils, to the synovial cavity and prevented articular and cartilage damage. Moreover, CXCL9(74–103) reduced neutrophil infiltration and neutrophil-dependent inflammation in the ears of the mice in a murine contact hypersensitivity model (265).

In contrast to other strategies that interfere with the chemokine-GAG interaction, the CXCL9-derived peptide, CXCL9(74–103), was not synthesized with the intention to specifically target the action of CXCL9, but rather focuses on the inhibition of the activity of a broader range of chemokines. Recently, two other groups reported the development of chemokine-derived GAG-binding peptides as well. McNaughton et al. synthesized CCL5-, CXCL8-, and CXCL12γ-derived peptides based on the knowledge of their GAG-binding regions (232). The peptides display sequence identity with the chemokines they are derived from. This approach aims at preserving the intrinsic specificity of the chemokine for the GAG by not interfering with hydrogen binding and Van der Waals interactions. The lead peptide pCXCL8-1, consisting of ten amino acids, was modeled based on the COOH-terminal α-helix of CXCL8, a region that mediates an important role in GAG binding. This peptide showed increased affinity for HS and DS in comparison with intact CXCL8 and displayed selectivity for HS over DS. Moreover, pCXCL8-1 competed with CXCL8 for binding to HS. In contrast to pCXCL8-1, a CXCL12γ-derived peptide failed to inhibit CXCL8-induced neutrophil migration, despite its high affinity for HS. This indicates that specificity plays a role in the interaction between the peptide and HS. Accordingly, the binding sites for the CXCL8- and the CXCL12γ-derived peptides on HS may differ. The peptide pCXCL8-1 was modified (NH_2_-terminal acetylation and COOH-terminal amidation) in order to protect it from proteolytic cleavage by exopeptidases upon administration *in vivo*. The resulting peptide pCXCL8-1_aa_ was tested in an *in vivo* murine model of antigen-induced arthritis. The number of neutrophils in the synovium was reduced. Furthermore, the inflammation, cellular exudate and hyperplasia were decreased upon treatment with pCXCL8-1_aa_. The peptide improved the arthritic score that is a measure of severity of disease in this mouse model (232). Martinez-Burgo et al. synthesized three different peptides derived from CXCL8: a COOH-terminal peptide (54–72) (wild-type peptide), a peptide (54–72) where the glutamic acid residue at position 70 was replaced with a lysine residue (E70K peptide) and a scrambled peptide consisting of the same amino acids as peptide 1 in a random order. Although only detectable at much higher concentrations compared to intact CXCL8, the three peptides, and the E70K peptide in particular, showed binding to heparin. The observed low-affinity binding of the peptides appeared to be dependent on charge. Furthermore, the peptides did not affect chemokine-GPCR binding. Only the E70K peptide showed the ability to inhibit CXCL8-mediated transendothelial migration of neutrophils (233).

### Dominant-Negative Chemokine Mutants

ProtAffin Biotechnologie AG developed a protein-based technology platform, also known as the CellJammer technology platform, in order to interfere with protein-GAG interactions via protein engineering. This approach enabled the generation of GAG-binding decoy proteins, namely dominant-negative mutant proteins. On the one hand, these proteins are characterized by increased GAG-binding affinity (= dominant mutations). On the other hand, dominant-negative mutant proteins display impaired receptor binding/activation (= negative mutations) (266–268). In order to improve GAG-binding affinity, non-crucial amino acids in the GAG-binding domain of the wild-type protein were substituted with basic amino acids. This resulted in an increase of the electrostatic component of the protein-GAG interaction. Furthermore, to disrupt the bioactivity of the wild-type protein, amino acids responsible for natural chemokine-receptor interactions are either substituted with alanine residues or deleted (266). The strategy relies on the intrinsic selectivity of the GAG-binding protein for its specific GAG epitope. Consequently, dominant-negative mutant proteins that bind with higher affinity to the GAG have the ability to displace their wild-type counterpart protein from the specific GAG target sequence. With this mode of action, dominant-negative mutant proteins antagonized protein-GAG interactions (266, 267).

A series of CXCL8 mutants was engineered by site-directed mutagenesis. Subsequently, by using a combinatorial approach consisting of different techniques, the affinities of the different mutants for HS were assessed. The pro-inflammatory human CXCL8 was designed toward an anti-inflammatory dominant-negative CXCL8 mutant. On the one hand, four non-crucial amino acids of human CXCL8 were replaced with basic lysine residues in order to knock-in high GAG-binding affinity. On the other hand, the six NH_2_-terminal amino acids of human CXCL8, including the ELR motif, were deleted in order to knock-out the binding to its two GPCRs, namely CXCR1 and CXCR2. This resulted in the mutant PA401 or CXCL8[Δ6F17KF21KE70KN71K] that was selected from the series of dominant-negative CXCL8 mutants as it showed the best interaction profile with HS (266, 269, 270). Moreover, complete knocked-out CXCR1 and CXCR2 activity was observed in this mutant (269, 271). The wild-type chemokine that is displaced by the dominant-negative chemokine mutant, may still activate leukocytes. However, since the endothelial contact with GAGs is disrupted, transmigration of the chemokine should not take place (271). In addition, other chemokines than CXCL8 can be displaced from GAGs by PA401 as well. PA401 had the ability to displace CCL2 from GAGs with a similar IC50 value in comparison with CXCL8. Furthermore, CXCL10, CXCL12, CCL11 and other chemokines were displaced by PA401 as well, although they were characterized by higher IC50 values (267, 271–273). PA401 had the ability to inhibit CXCL8-induced neutrophil migration (reduction by 75%) in a transendothelial migration assay (273).

The anti-inflammatory activity of PA401 was evaluated in several disease models. Treatment with PA401 reduced renal ischemia-reperfusion injury in a rat model. More specifically, proximal tubular damage was reduced and a decreased number of infiltrating granulocytes was observed. Furthermore, PA401 reduced acute allograft damage in a rat kidney transplantation model. PA401 treatment lowered glomerular infiltration of monocytes and CD8^+^ T cells. In addition, tubular interstitial inflammation and tubulitis, which is an indication of acute allograft rejection, were diminished as well. Moreover, the highest dose of PA401 improved glomerular and vascular rejection (271). In a murine model of acute inflammation, PA401 dose-dependently inhibited neutrophil recruitment to the knee cavity after intra-articular injection with murine CXCL1. Thus, PA401 showed anti-inflammatory activity in an inflammation model that was induced by a murine functional homolog of CXCL8. Furthermore, PA401 treatment was evaluated in a murine model of antigen-induced arthritis and resulted in inhibition of leukocyte adhesion, diminished neutrophil recruitment and inflammation-related hypernociception (269). Moreover, PA401 had the ability to disrupt the CXCL8-GAG interaction in bronchoalveolar fluid samples from patients with cystic fibrosis. As a consequence, the release of CXCL8 from the GAGs rendered the chemokine susceptible to proteolytic degradation, resulting in reduced migration of neutrophils (274). PA401 was also tested as a novel therapeutic approach in two murine models characterized by neutrophilic lung inflammation. In a murine model of LPS-induced lung inflammation, the administration of PA401 reduced the total number of cells and the number of neutrophils in bronchoalveolar lavage (BAL). Furthermore, PA401 had the ability to decrease lung congestion and inflammatory cells in the lung tissue (230) and normalized plasma inflammatory markers (275). In a murine model of tobacco smoke-induced lung inflammation, PA401 treatment showed broad anti-inflammatory activities, including reduced inflammatory cells and soluble inflammatory markers. A reduction in the number of neutrophils, macrophages, lymphocytes and epithelial cells in BAL was observed (230). Furthermore, PA401 treatment was tested in a murine model of urinary tract infection and resulted in decreased recruitment of neutrophils to the urine. Normalization of the tissue architecture could be observed in mice treated with PA401 when inspecting histopathology sections of renal micro-abscesses (273). In a murine model of bleomycin-induced pulmonary fibrosis, a dose-dependent decrease in total cell counts and neutrophils was observed in BAL samples upon PA401 treatment. Moreover, decreased levels of CXCL1 were detected in lung tissue (273). In an experimental autoimmune uveitis model in rats, treatment with PA401 influenced severity and incidence of disease. The mean maximal clinical disease scores were decreased after both pre-symptomatic and symptomatic treatment with PA401. Furthermore, slightly less retinal destruction was observed after PA401 treatment (273).

A phase I first-in-human clinical trial (NCT01627002) was performed to examine the safety, tolerability, immunogenicity and pharmacokinetics of PA401 in healthy volunteers. Moreover, the effect of PA401 on lung inflammation following an LPS challenge was investigated in this study as well.

The CellJammer approach was applied to develop a second dominant-negative chemokine mutant, namely a CCL2/MCP-1-based decoy protein named PA508. Met-CCL2[Y13AS21KQ23R] was selected out of four novel CCL2 mutants as it showed both the highest affinity for HS and knocked-out CCR2 activity. The serine and glutamine residues at position 21 and 23 respectively, are characterized by solvent-exposed areas above 30% and are located close to the GAG-binding site of the chemokine. Consequently, these amino acid residues were substituted with basic amino acids in order to increase the GAG-binding affinity. Moreover, these mutations were beneficial for the disruption of receptor activation, considering the partial overlap between the GAG-binding and receptor activation sites in CCL2. Since the tyrosine residue at position 13 is a key residue for receptor signaling, it was replaced by an alanine residue, thereby abrogating CCR2 activation and signaling. The NH_2_-terminal methionine residue, originating from recombinant protein synthesis in *E. coli*, was not removed as it turned out to increase the binding affinity to heparin and decreased binding affinity for CCR2 (276). PA508 has a remarkable specificity for CCL2, as it did not influence transendothelial migration of monocytic cells induced by chemokines such as CCL5 and CXCL1. Moreover, this specificity was confirmed *in vivo* as PA508 showed no antagonistic activity in CCR2^−/−^ mice, indicating that PA508 specifically targeted the CCL2-CCR2 axis (277).

The anti-inflammatory activity of PA508 has been demonstrated in several *in vivo* models. The administration of PA508 resulted in a mild ameliorating effect in rat experimental autoimmune uveitis. PA508-treated rats were protected from the development of severe inflammation of the inner eye (276). Furthermore, treatment with PA508 reduced the influx of leukocytes in a murine air pouch model of TNF-α-induced leukocyte recruitment. In a mouse model of wire-induced neointimal hyperplasia, PA508 reduced neointimal plaque area in wire-injured arteries. In addition, this reduction was associated with diminished macrophage infiltration. The smooth muscle cell content in the neointima was increased upon treatment. Thus, treatment with PA508 reduced neointima formation and resulted in a more stable, less inflammatory plaque phenotype (277). Administration of PA508 attenuated myocardial ischemia-reperfusion injury in mice. Treatment preserved heart function and reduced myocardial infarction size. The latter resulted from PA508-mediated inhibition of myocardial macrophage-related inflammation and reduction of myofibroblast and collagen content (277). In a murine model of zymosan-induced peritonitis, administration of PA508 reduced infiltration of a pro-inflammatory subset of monocytes (Gr1 and F4/80 double positive), whereas the number of peritoneal macrophages was not affected (278). PA508 treatment showed also promising results in murine experimental autoimmune encephalomyelitis. PA508-treated mice showed a delayed disease onset and an overall better clinical score. Furthermore, mice that received PA508 demonstrated decreased maximal disease severity, preserved body weight and increased survival. Treatment with PA508 resulted in a reduction of inflammatory cell infiltrates in the spinal cord and the cerebellum, as observed during histological analysis and demyelination was reduced (278).

Chemokines and their respective chemokine mutants are characterized by a short serum half-life. In order to increase the bioavailability and thus prolong the serum half-life of PA508 for chronic indications, a novel mutant CCL2-human serum albumin (HSA) fusion protein was designed. Considering the steric influence of HSA, a diminished GAG-binding affinity of this new mutant CCL2-HSA chimera was expected. Therefore, a series of novel mutants with additional basic amino acids was developed. Eventually, the fusion of the selected CCL2 mutant with HSA resulted in HSA(C34A)-(Gly)_4_Ser-Met-CCL2[Y13AN17KS21KQ23KS34K] that demonstrated high and selective GAG-binding affinity and improved stability (279).

The CellJammer technology was used also to generate CCL5-based decoy proteins. Initially, ten CCL5 mutants were developed. All mutants retained their NH_2_-terminal methionine residue that resulted from bacterial expression, rendering them into functional receptor antagonists. Two mutants, A22K and H23K, were selected since they demonstrated highest stability and improved GAG binding. Their GAG-binding affinity was increased by engineering an extended three-dimensional GAG-binding epitope in addition to the linear ^44^RKNR^47^ GAG-binding domain. Both mutants displayed completely abrogated chemotaxis on monocytes, due to their extra NH_2_-terminal methionine. Treatment with the H23K mutant in rat autoimmune uveitis led to earlier recovery from ocular inflammation, whereas treatment with the A22K mutant did not demonstrate any anti-inflammatory effect. Brandner et al. hypothesized that the minor therapeutic effect of both mutants could be due to a deficiency in GAG-induced oligomerization caused by these mutations. The H23K mutant exhibited higher GAG-binding affinity and partially retained the ability to form GAG-induced oligomers, which could explain its observed therapeutic effect in comparison with the A22K mutant of CCL5 (280).

In addition, a CXCL12α-based decoy protein was developed. Deletion of the first eight amino acids of the chemokine already resulted in completely impaired chemotaxis. Furthermore, the amino acid residues at positions 29 and 39 were mutated as these positions are important for the first receptor binding step and for GAG binding. This resulted in the dominant-negative CXCL12α mutant CXCL12α[Δ8L29KV39K], which demonstrated impaired CXCR4 signaling in combination with increased and specific GAG affinity. The effect of this mutant was assessed in an *in vivo* murine breast cancer seeding model. Treatment with the CXCL12α mutant inhibited migration of cancer cells, resulting in a decreased number of liver metastases (281).

### The Spiegelmer NOX-A12

NOX-A12 is an aptamer inhibitor of CXCL12 that can be classified more specifically as a Spiegelmer. Mirror-image aptamers or Spiegelmers are synthetic oligonucleotides that are composed of non-natural L-nucleotides. Since naturally occurring enzymes are stereoselective for nucleic acids in the D-configuration, Spiegelmers are protected from nucleolytic cleavage, implying a native biological stability (282, 283). Spiegelmers can be selected *in vitro* to bind a wide variety of molecular targets with high affinity and specificity (283, 284). Binding to a specific molecular target relies on the three-dimensional structure of a Spiegelmer, which is determined by its nucleotide sequence (283, 285). This interaction via three-dimensional structures is similar to antibody–antigen binding. Moreover, aptamers are functionally comparable to antibodies regarding binding affinity and specificity for their targets. They display advantages relative to antibodies, including smaller size, higher stability, fast *in vitro* chemical production, wide spectrum of potential targets and non-immunogenicity (285, 286).

The Spiegelmer NOX-A12 or olaptesed pegol is a 45-nucleotide long L-RNA aptamer that interferes with chemokine-GAG interactions (228, 283, 286). NOX-A12 was developed to bind and antagonize CXCL12 in the tumor microenvironment and for cell mobilization (228, 287). CXCL12 plays a critical role in physiological and pathological processes such as embryogenesis, haematopoiesis, angiogenesis and inflammation (200, 288). This chemokine functions by interaction with its two receptors CXC chemokine receptor 4 (CXCR4) and atypical chemokine receptor 3 (ACKR3) (200). NOX-A12 bound its molecular target with subnanomolar affinity (287). Because of its small size, NOX-A12 was rapidly excreted through renal filtration (285). However, in order to extend its plasma half-life, NOX-A12 was conjugated to a branched polyethylene glycol (PEG) moiety of 40 kDa (PEGylated) (289).

CXCL12 also plays a critical role in the pathogenesis of chronic lymphocytic leukemia (CLL). This chemokine is important for migration and retention of CLL cells in tissues such as the BM. Bone marrow stromal cells (BMSCs) constitutively secrete CXCL12 and consequently attract CLL cells into the supportive microenvironment via activation of the CXCR4 receptor that is expressed on these leukemic cells. CXCL12 is presented to CXCR4 by binding to GAGs on the cell surface or ECM. In the tissue microenvironment, the CLL cells are protected from cytotoxic drugs and they receive survival signals via several factors, including CXCL12. Interfering with the cross-talk between CLL and stromal cells in order to eliminate CLL cells from the protective microenvironment and sensitize CLL cells to conventional therapy can be done by targeting CXCL12/CXCR4 signaling (228, 229).

Hoellenriegel et al. investigated the effect of NOX-A12 on the migration of CLL cells and drug sensitivity. Moreover, they more thoroughly investigated the mechanism of action of NOX-A12 and revealed its ability to compete with GAGs for CXCL12 binding. SPR measurements were performed with immobilized heparin and CXCL12 was injected together with NOX-A12. The latter competed with the immobilized heparin for binding to CXCL12, resulting in detachment of heparin-bound CXCL12. Consequently, the binding site of NOX-A12 on CXCL12 was presumably close to or overlaps with the heparin-binding site of CXCL12. Hence, the mode of action of NOX-A12 involved competition with heparin for binding to CXCL12 and explained the detachment of CXCL12 from extracellular GAGs (228).

Another Spiegelmer that binds to and neutralizes a chemokine, i.e. Emapticap pegol or NOX-E36, bound to CCL2, also known as monocyte chemoattractant protein (MCP)-1 (283, 290). However, to the best of our knowledge, it has not been explored or proven whether the mechanism of action of the latter Spiegelmer is similar to the one of NOX-A12. Accordingly, NOX-E36 will not be further discussed in this review.

NOX-A12 has been used in a variety of different disease models, ranging from chronic kidney disease to several types of cancer. An overview of studies that report treatment with NOX-A12 in different (animal) models and clinical trials can be found in Table 2. Currently, NOX-A12 is being tested alone and in combination with the immune checkpoint inhibitor Pembrolizumab in an ongoing phase I/II clinical trial of metastatic pancreatic and colorectal cancer (NCT03168139). In addition, the recruitment of patients with newly diagnosed glioblastoma/glioma has started in order to initiate a phase I/II clinical trial to evaluate combination treatment of NOX-A12 and radiotherapy^1^.

**Table 2:** Overview of the use of NOX-A12 as a treatment strategy in different preclinical models and clinical trials.

**Species**	**Disease model**	**Treatment**	**Result**	**References**
Mouse	Proliferative lupus nephritis	NOX-A12 + NOX-E36	- ↓ Proteinuria - ↓ renal excretory failure - ↓ immune complex glomerulonephritis - ↓ potentially reversible and irreversible structural kidney injury - ↓ expansion of lymphocytes and plasma cells in spleen	(291)
Mouse	Chronic kidney disease	NOX-A12	- ↑ podocyte counts - ↓ proteinuria - ↓ glomerular lesions - ↓ renal dysfunction	(292)
Mouse	Type 2 diabetes, diabetic nephropathy	NOX-A12	- ↓ glomerulosclerosis - ↑ number of podocytes - Prevention of proteinuria - Improvement of tubular damage and peritubular vasculature density	(287)
Mouse	Type 2 diabetes, diabetic nephropathy	NOX-A12 + NOX-E36	- ↓ glomerulosclerosis - ↑ number of podocytes - Prevention of proteinuria - ↓ number of glomerular leukocytes - Protective effect on GFR decline	(293)
Mouse	Islet transplantation	NOX-A12 + mNOX-E36	- Improved islet survival and function - ↓ recruitment of inflammatory monocytes in the graft site	(294)
Mouse	Type 1 diabetes	NOX-A12	- ↓ inflammation-mediated islet destruction	(294)
Mouse and cynomolgus monkey	HSC mobilization	NOX-A12	Mobilization of leukocytes and HSCs into peripheral blood	(289)
Human (phase I: first-in-human)	HSC mobilization	NOX-A12	- Benign safety profile - dose-dependent mobilization of leukocytes and HSCs into peripheral blood	(289)
Human CLL cells, human lymphoid cell lines, murine stromal cell lines	CLL	NOX-A12	- Inhibition of CXCL12-induced chemotaxis of CLL cells - ↑ CLL migration underneath a confluent layer of BMSCs - release of CXCL12 from cell-surface-bound GAGs - competition with heparin for binding to CXCL12 - Sensitization of CLL cells toward cytotoxic agents in BMSC cocultures	(228, 229)
Human (phase IIa)	Relapsed/refractory CLL	NOX-A12 + bendamustine + rituximab	- Effective mobilization of CLL cells (for at least 72 h) - Combination therapy generally well tolerated - High ORR of 86% (with 11% CR) - Median PFS of 15.4 months in ITT population - 3-year overall survival rate of >80% in ITT population	(295)
Mouse	CML	NOX-A12 + nilotinib	↓ leukemia burden	(296)
Mouse	(MM)	NOX-A12	- Microenvironment less receptive for MM cells - ↓ MM cell homing and growth - Inhibition of MM tumor progression - ↑ survival - ↓ MM cell bone metastases - chemosensitization of MM cells to bortezomib	(297)
Human (phase IIa: first-in-patient)	Relapsed/refractory MM	NOX-A12 + bortezomib-dexamethasone	- Effective mobilization of myeloma cells (for at least 72 h) - ↑ clinical activity of bortezomib-dexamethasone	(298)
MM cell lines	MM	NOX-A12 + carfilzomib	No increased cytotoxic effect compared with carfilzomib alone	(299)
Rat	Glioblastoma multiforme	NOX-A12	- Inhibition or delay of tumor recurrences following irradiation - prolongation of median life span	(300)
Mouse, rat	Glioblastoma multiforme	NOX-A12 + anti-VEGF (bevacizumab or B-20)	- ↑ survival - ↓ tumor associated macrophages - Potentiation antitumor efficacy of anti-VEGF	(301)
Tumor-stroma spheroids, mouse	Colorectal cancer	NOX-A12 + anti-PD-1 therapy	- ↑ infiltration of CD8^+^ T cells, CD4^+^ T cells and NK cells into spheroids - ↑ T cell activation in spheroids - ↓ tumor growth - ↑ efficacy of anti-PD-1 therapy	(302)
Rat	Idiopathic pulmonary arterial hypertension	NOX-A12	- ↓ perivascular CD68^+^ macrophages, CD3^+^ T cells, mast cells - ↓ pulmonary vascular remodeling - Improvement of haemodynamics and right heart hypertrophy	(303)
Mouse	Chronic allograft vasculopathy	NOX-A12	- ↓ neointima formation - ↓ expression of pro-fibrotic inflammatory cytokines - ↓ infiltrating CD3^+^ cells	(304)
Mouse	Retinal degradation	NOX-A12 + intravitreal injection of CXCL12	- ↑ homing of bone marrow-derived stem cells into the damaged retina - ↑ visual function	(305)

*GFR, glomerular filtration rate; HSC, haematopoietic stem and progenitor cell; CLL, chronic lymphocytic leukemia; ORR, overall response rate; CR, complete remission; PFS, progression-free survival; ITT, intent-to-treat; CML, chronic myeloid leukemia; MM, multiple myeloma; VEGF, vascular endothelial growth factor*.

## Conclusion

Evidence is accumulating that the chemokine-GAG interaction may be an interesting target for the inhibition of inflammation. During an exaggerated inflammatory response, the aim would be to reduce inflammation, but not to completely inhibit the inflammatory response. The use of compounds that interfere with the chemokine-GAG interaction could be feasible in this case. On the contrary, complete inhibition of inflammation is probably not feasible, and also not desired. Focusing on the chemokine-GAG interaction is often associated with lower specificity and lower efficiency in comparison with targeting chemokine-receptor interactions. However, these characteristics might actually be beneficial if a reduction of excess inflammation is the objective.

Various therapeutic approaches, including CKBPs (viral, tick, and human), chemokine-derived GAG-binding peptides, dominant-negative chemokine mutants and the Spiegelmer NOX-A12, were discussed in this review and they all showed promising results in inhibiting chemokine-GAG interactions, independent of their specific mechanisms of action. As some of these therapeutic approaches are currently in an initial stage of research, whereas other approaches are already in clinical trials, it is delicate to compare their effect and predict their future applications. In order to generate novel therapeutics that reduce inflammation by inhibiting the chemokine-GAG interaction, further research is required to elucidate the effect on inflammation in different *in vivo* models of disease and to explore thoroughly the range of therapeutic applications.

## Author Contributions

HC and VV wrote the initial version of the manuscript as part of their Ph.D. thesis under supervision of PP. The manuscript was finally revised by all authors.

### Conflict of Interest

The authors declare that the research was conducted in the absence of any commercial or financial relationships that could be construed as a potential conflict of interest.

## Footnotes

^1^https://www.noxxon.com/index.php?option=com_content&view=article&id=21&Itemid=478.
